# A unifying Bayesian account of contextual effects in value-based choice

**DOI:** 10.1371/journal.pcbi.1005769

**Published:** 2017-10-05

**Authors:** Francesco Rigoli, Christoph Mathys, Karl J. Friston, Raymond J. Dolan

**Affiliations:** 1 The Wellcome Trust Centre for Neuroimaging, UCL, 12 Queen Square, London, United Kingdom; 2 City, University of London, Northampton Square, London, United Kingdom; 3 Scuola Internazionale Superiore di Studi Avanzati (SISSA), Trieste, Italy; 4 Max Planck UCL Centre for Computational Psychiatry and Ageing Research, London, United Kingdom; 5 Translational Neuromodeling Unit (TNU), Institute for Biomedical Engineering, University of Zurich and ETH Zurich, Zurich, Switzerland; New York University, UNITED STATES

## Abstract

Empirical evidence suggests the incentive value of an option is affected by other options available during choice and by options presented in the past. These contextual effects are hard to reconcile with classical theories and have inspired accounts where contextual influences play a crucial role. However, each account only addresses one or the other of the empirical findings and a unifying perspective has been elusive. Here, we offer a unifying theory of context effects on incentive value attribution and choice based on normative Bayesian principles. This formulation assumes that incentive value corresponds to a precision-weighted prediction error, where predictions are based upon expectations about reward. We show that this scheme explains a wide range of contextual effects, such as those elicited by other options available during choice (or within-choice context effects). These include both conditions in which choice requires an integration of multiple attributes and conditions where a multi-attribute integration is not necessary. Moreover, the same scheme explains context effects elicited by options presented in the past or between-choice context effects. Our formulation encompasses a wide range of contextual influences (comprising both within- and between-choice effects) by calling on Bayesian principles, without invoking *ad-hoc* assumptions. This helps clarify the contextual nature of incentive value and choice behaviour and may offer insights into psychopathologies characterized by dysfunctional decision-making, such as addiction and pathological gambling.

## Author summary

Research has shown that decision-making is dramatically influenced by context. Two types of influence have been identified, one dependent on options presented in the past (between-choice effects) and the other dependent on options currently available (within-choice effects). Whether these two types of effects arise from similar mechanisms remain unclear. Here we offer a theory based on Bayesian inference which provides a unifying explanation of both between and within-choice context effect. The core idea of the theory is that the value of an option corresponds to a precision-weighted prediction error, where predictions are based upon expectations about reward. An important feature of the theory is that it is based on minimal assumptions derived from Bayesian principles. This helps clarify the contextual nature of incentive value and choice behaviour and may offer insights into psychopathologies characterized by dysfunctional decision-making, such as addiction and pathological gambling.

## Introduction

Standard theories of decision-making assume that the incentive value of an option should be independent of options presented in the past and options available during choice [1–4]. These theories are fundamentally challenged by empirical evidence showing that expectations (derived from past experience) about upcoming options change value attribution and choice behaviour [5–14]. For example, in a series of recent experiments from our lab [8–10], participants made choices in blocks (i.e. contexts) associated with one of two distinct, but partially overlapping, reward distributions. Participants’ choices were consistent with attributing a larger incentive value to rewards (common to both contexts) in blocks associated with low compared to high average reward. In other words, the incentive value of a reward increased when the average was lower. In addition to the average reward of a context, evidence from a similar task indicated that reward variance within a given context also exerts an influence on incentive value [11]. These findings highlight contextual effects exerted by expectations about options (induced, for example, by options available during previous choices); namely, *between-choice* contextual effects.

**Fig 1:**
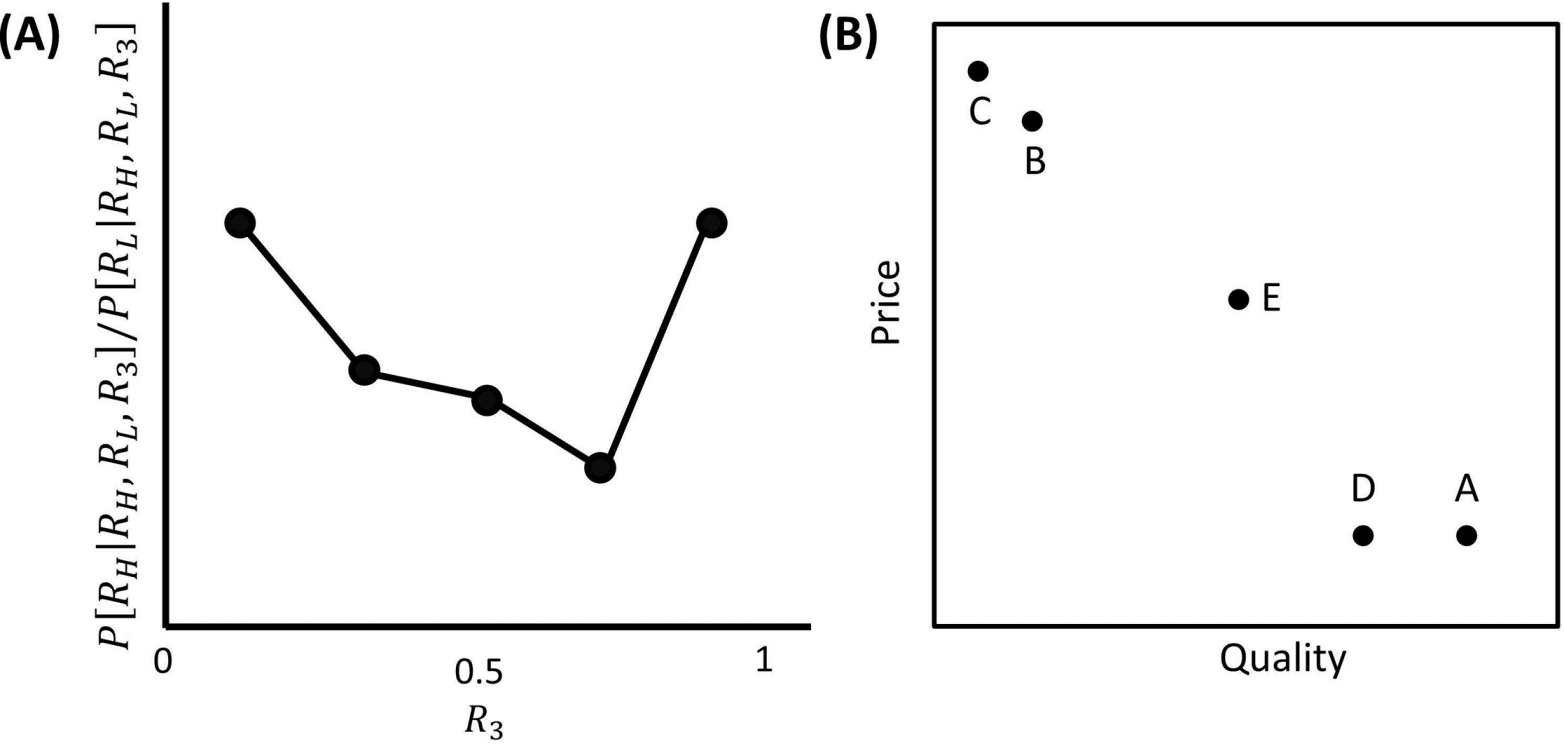
**A:** Empirical evidence concerning contextual effects elicited by multiple options available during choice (within-choice context effects). **A:** Single-attribute choice, where there is no need to integrate multiple attributes to make a decision. Here a better target option associated with reward *R*_*H*_ and a worse target option associated with reward *R*_*L*_ are available together with a third option associated with reward *R*_3_. The graph plots empirical findings [6] in terms of the ratio between the probability of choosing the better target option (*P*[*R*_*H*_|*R*_*H*_,*R*_*L*_,*R*_3_]) and the probability of choosing the worse target option (*P*[*R*_*L*_|*R*_*H*_,*R*_*L*_,*R*_3_]) as a function of the (normalized) reward of a third options *R*_3_ (see Fig 5C in [6]). **B:** Multiattribute choice, where multiple attributes need to be integrated to make a decision. Here, we consider the difference in choice probability between a high-quality and high-price car A and a low-quality and low-price car B. Although during binary choice this difference is zero, empirical evidence has shown this difference can be non-zero when a third option is also available. A similarity effect favours car A over car B when a low-quality and low-price car C (similar to car B) is available. An attraction effect favours car A over car B when a medium-quality and high-price car D is available. A compromise effect consists in favouring a medium-quality and medium-price car E over both car A and car B during choices in which all three cars are available, despite the fact these cars are equally chosen when they are available in pairs during binary choices.

In addition, the empirical literature has highlighted contextual influences elicited by options available during choice; namely, *within-choice* context effects [6, 15–20]. Standard theories of decision-making assume that the incentive value of an option should be independent of other options available during choice [1–4]. This implies that the choice proportion between two options, comprising a more valuable and a less valuable option, should be unaffected by the introduction of a third [2]. However, a recent study [6] has shown that this choice proportion follows a U-shape function, which diminishes as the value of a third option approaches the value of the target options–and starts increasing thereafter (Fig 1A). This is hard to reconcile with standard theories and represents a form of within-choice context effect, whereby the value of an option is affected by other options available during choice.

In this task, it is unnecessary to compare options across different attributes (single-attribute decisions; [6]). However, other forms of within-choice context effect have been observed when options are defined by the same set of attributes that have to be traded of against each other (multiattribute decisions; [15–20]. For example, consider a binary choice between a high-quality and expensive car A versus a low-quality and cheap car B (Fig 1B). Imagine the values of the attributes are such that an agent is indifferent about the two options (i.e., the higher price of car A is exactly compensated by its quality), resulting in the same probability of choosing options A and B. What happens if a third option is also available? Standard models (based on the assumption that values are independent of other options) predict that the choice probability difference will remain zero, independent of a third option. However, empirical data highlight a so-called *similarity effect* [20–23], whereby preference for an option over a second option–which is equally preferable during binary decisions–increases if a third option is available that is similar to the second option (Fig 1B). In our example, the choice probability difference between car A and car B will be positive when a third low-quality and cheap (similar to car B) car C is also available. A form of influence called the *attraction effect* [15, 24–26] has also been found with the availability of a third option that is characterized by a low score for one attribute and an intermediate score for the other (Fig 1B). The presence of such a third option favours the option with a high score for the attribute for which the third option has an intermediate score. In our example, the choice probability difference between car A and car B will be positive when a third medium-quality and expensive car D is also available. Finally, empirical data are consistent with a so-called *compromise effect* [17, 25, 27]. This applies when the choice set includes two options scoring high in one attribute and low in another plus a third option characterized by intermediate scores for both attributes. While the three options are equally preferred (i.e., are chosen an equal amount of times) if presented in pairs during binary choices, when they are all available, a preference for the option characterized by intermediate scores is observed (Fig 1B). For instance, although during binary choices an average-price and average-quality car E is not preferred over car A or over car B, car E will be favoured when presented together with both car A and car B.

Several explanations have been proposed to account for contextual effects on incentive value and choice, with most models focusing on within-choice context effects during multiattribute decisions [16–20, 27, 28]. Other theories have been proposed to explain between-choice context effects [29–31], and disregard within-choice effects. We are aware of a single attempt to encompass both between-choice and within-choice effects, though restricted to non-multiattribute decisions for the latter type of effects [6]. Whether models developed to explain a certain class of context effects generalise to other effects remains unclear–and a unifying account encompassing all known context effects is lacking. Developing a parsimonious account would represent an important theoretical advance, as it would explain diverse empirical phenomena with the same underlying principles.

The goal of the present paper is to describe a unifying theory, referred to as Bayesian model of context sensitive value (BCV) that explains between-context and within-context effects, in single and multiattribute decisions. This theory represents a generalization of a recent model developed to explain between-choice contextual effects [11]. The key idea is that agents build a generative model of reward within a context and, every time a new reward or option is presented, use Bayesian inference to invert this model to form a posterior belief about the underlying reward distribution. Incentive value is computed during this belief update and corresponds to a precision-weighted reward prediction error. The advantage of this theory relies on its grounding upon simple normative principles of Bayesian statistics. In addition, the model can explain between-choice context effects [8–11] and makes specific predictions that have been confirmed empirically.

In brief, BCV proposes that the incentive value of a stimulus (or option) corresponds to the change in reward expected (in any given context) when the stimulus is presented. This makes precise predictions about choice under ideal (Bayesian) observer assumptions (with a minimal number of free parameters). Crucially, predictions include specific forms of context effects, and raise a question of whether these predicted effects are consistent with empirical findings.

In this paper, we applied BCV to multi-alternative choice (considering both single and multiattribute decisions) and ask whether the model predicts the context-effects found empirically. We first present a theoretical extension of BCV applicable to decisions in which multiple options are available and can be characterized by multiple attributes. We show that predictions derived from the model are remarkably similar to empirical findings on within-choice contextual effects, both during non-multiattribute and multiattribute decisions. We next review BCV in relation to between-choice context effects and describe how the model can also explain these empirical findings. On this basis, we offer the model as a principled description of between and within-choice context effects.

## Results

### Within-choice context effects

The idea behind BCV is to establish a link between theories of value and normative accounts of brain functioning based on Bayesian statistics [32–37]. The Bayesian brain framework rests on the idea that an agent builds a model of the processes generating sensory cues. The generative model comprises a set of random variables (i.e., hidden states or causes of sensory outcomes) and their causal links (i.e., probabilistic contingencies). The variables can be separated into hidden and observable variables, the former representing the latent causes of observations, and the latter representing sensory evidence or cues. Sensory evidence conveyed by observable variables is combined with prior beliefs about hidden causes to produce a posterior belief about the causes of observations. The application of this logic has proved effective in explaining several empirical phenomena in perception [32–37]. For instance, psychophysical data indicate that human perception depends on integrating different perceptual modalities (e.g., visual and tactile) in a manner consistent with Bayesian principles [38], where evidence is weighted by the precision of sensory information. Furthermore, process theories that mediate Bayesian inference (e.g., predictive coding) have a large explanatory scope in terms of neuroanatomy and physiology [39].

Inspired by a recent framework that conceptualises planning and choice as active inference [40–45], our core proposal is that Bayesian inference drives the attribution of incentive value to reward, and this in turn determines choice.

In a previous work, we have developed a version of BCV applicable to conditions where past options elicit context effects by shaping expectancies before a reward is presented ([11]; see below). However, our previous formulation did not consider conditions where multiple options (potentially characterized by multiple attributes) are available. Here we generalize BCV to encompass conditions in which multiple options are available and options can be characterized by multiple attributes. We define a multi-attribute *option u*_*n*_ (e.g., car A or car B) as a contract that yields *reward amount R*_*i*,*n*_ relative to each attribute *i* (e.g., price or quality):

$$u_{n}={\left\{R_{i,n}\right\}}_{i=1,\ldots ,I_{n}}\;with\;R_{i,n}\in R$$ (1)

An option set *u* is the set of options currently available:

$$Set\;{u=\{u_{n}\}}_{n=1,\ldots ,N}\;\mathrm{with}\;u_{n}an\;option\;\forall \;n$$ (2)

The expected value (EV) of an option *u*_*n*_ corresponds to:

$$R_{u_{n}}=\sum_{i}R_{i,n}$$ (3)

**Fig 2:**
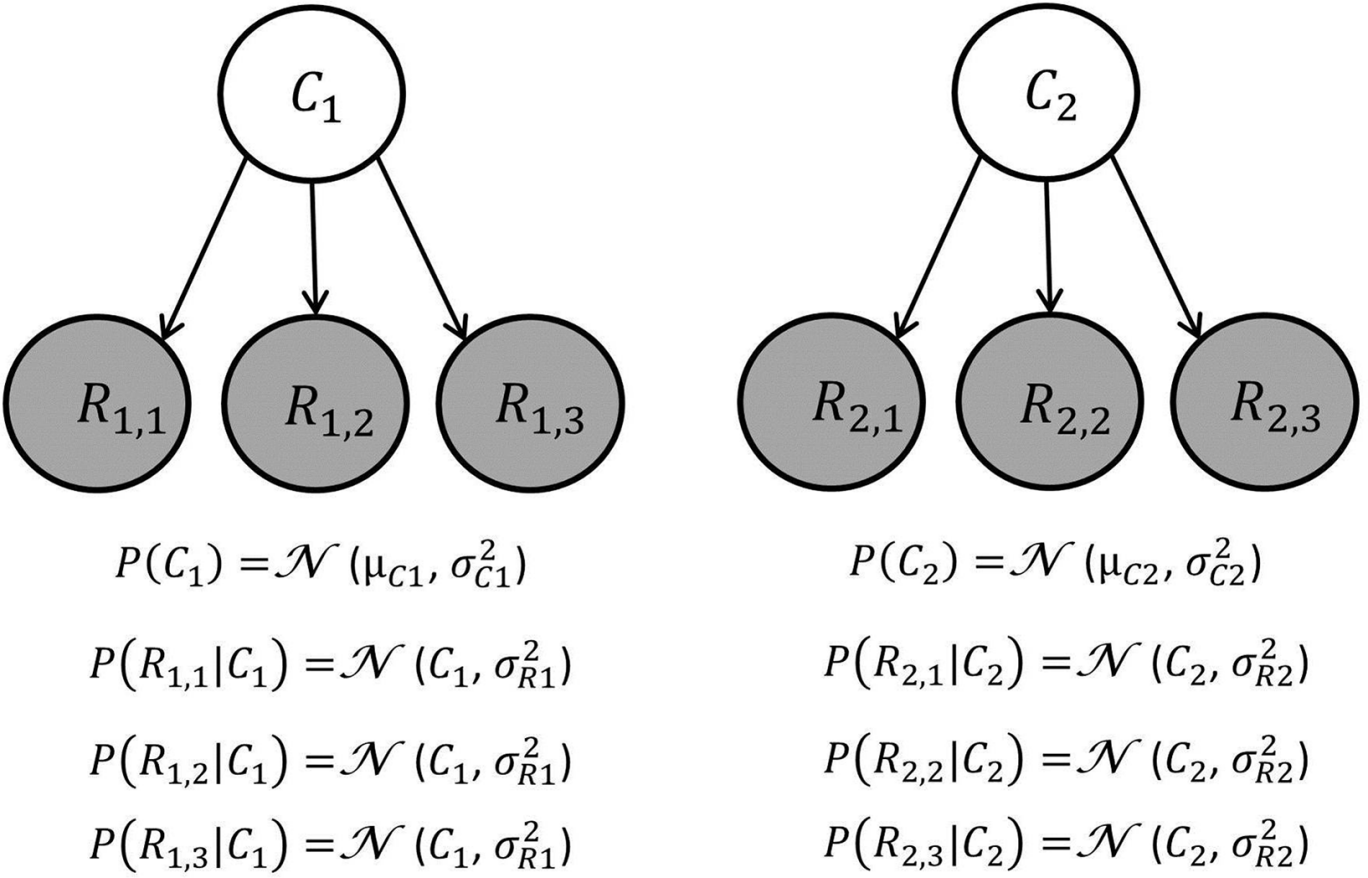
Example of a basic generative model underlying BCV. This is a directed acyclic graph or Bayesian network. Circles represent random variables (shaded and white circles refer to observed and non-observed variables respectively). An arrow denotes a conditional dependence–in which one random variable supplies the mean of the probability distribution of its children. For each attribute i, a hidden variable *C*_*i*_ represents the belief about the average reward amount across options for the attribute i. This generates the mean for Gaussian observable variables *R*_*i*,*n*_, corresponding to reward amounts associated with options available during choice. In this example, three options are available and options are characterized by two attributes. Note that attributes are independent in the generative model, as there is no arrow connecting variables associated with different attributes. Inverting this model, given observations, furnishes posterior beliefs over the mean reward amount across options for each attribute i. This inference is performed sequentially integrating one reward amount observation at each inference step. When a reward observation is considered, its incentive value is conceived as (precision-weighted) reward prediction error.

For example, the total reward for car A is equal to the reward associated with price plus the reward associated with quality. BCV assumes that an agent builds a generative model of the reward amounts *R*_*i*,*n*_ (Fig 2). Specifically, an agent believes that, for each attribute *i*, reward amounts *R*_*i*,*n*_ across options are sampled from the same population. To distinguish among attributes, we assume that an agent believes that an independent population of reward amounts is associated to each attribute. For example, if two attributes characterize options, two independent populations of reward amounts are considered by the agent (Fig 2).

Formally, for each attribute *i*, the average of the population of the reward amounts *R*_*i*,*n*_ is represented by a random variable *C*_*i*_, which is assumed to be sampled from a Gaussian distribution with prior mean *μ*_*Ci*_ and uncertainty (variance) $\sigma_{Ci}^{2}$:

$$C_{i}∼N(\mu_{Ci},\sigma_{Ci}^{2})$$ (4)

The agent assumes that *μ*_*Ci*_ and $\sigma_{Ci}^{2}$ are known but that *C*_*i*_ is not directly observable and therefore needs to be inferred from observing the different instances of reward amounts *R*_*i*,*n*_ of options for the attribute *i*. This is realized in the generative model by treating *C*_*i*_ as a hidden cause of Gaussian variables *R*_*i*,*n*_ with mean *C*_*i*_ and uncertainty $\sigma_{Ri}^{2}$:

$$R_{i,n}∼N(C_{i},\sigma_{Ri}^{2})$$ (5)

On the basis of the generative model, for each attribute *i*, the agent can estimate ${\hat{C}}_{i}=N({\hat{\mu }}_{Ci|Ri},{\hat{\sigma }}_{Ci|Ri}^{2})$, namely the posterior belief about the variable *C*_*i*_ (i.e., the average reward amount relative to the attribute *i*; the hat symbol indicates estimates of unknown quantities), given the observation of all reward amounts of all options available for the attribute *i*, represented by the set ***R***_*i*_. In other words, an agent assumes that there is an average reward for each attribute which is unknown but can be estimated based on the reward amounts.

According to Bayes’ rule, the posterior belief of *C*_*i*_ can be calculated by considering the associated *R*_*i*,*n*_ sequentially in any order. We propose such sequential belief updating for BCV, even if options (and the associated reward amounts) are presented simultaneously, and we assume that the order of options considered is random (with potentially different orders for different attributes). For example, when three options characterized by two attributes are available (represented by *R*_1,1_, *R*_1,2_ and *R*_1,3_ for attribute one, and *R*_2,1_, *R*_2,2_ and *R*_2,3_ for attribute two), inference can involve computing, in order, *P*(*C*_1_|*R*_1,1_), *P*(*C*_1_|*R*_1,1_, *R*_1,3_) and *P*(*C*_1_|*R*_1,1_, *R*_1,2_ *R*_1,3_) for attribute one, and *P*(*C*_2_|*R*_2,3_), *P*(*C*_2_|*R*_2,1_, *R*_2,3_) and *P*(*C*_2_|*R*_2,1_, *R*_2,2_ *R*_2,3_) for attribute two. In the example above, an agent may consider first car A and next car B when estimating the average reward for price, and first car B and next car A when estimating the average reward for quality.

The rationale behind sequential belief updating is that the brain is equipped with a limited computational capacity, which precludes the instantaneous (and parallel) evidence accumulation, and hence requires the processing of one option after another. A similar evidence accumulation process is implicit in some theories of perceptual and value-based decision-making (e.g., [16, 46, 47]). Below, we will show that this evidence accumulation, in the form of sequential Bayesian belief updating, endows agents with the right sort of sensitivity to context. Formally, if *R*_*i*,*n*_ is the reward amount considered first during belief updating, in relation to attribute *i*, the posterior mean ${\hat{\mu }}_{Ci|R_{i,n}}$ is [48]:

$${\hat{\mu }}_{Ci|R_{i,n}}=\mu_{Ci}+\frac{\sigma_{Ci}^{2}}{\sigma_{Ci}^{2}+\sigma_{Ri}^{2}}(R_{i,n}-\mu_{Ci})$$ (6)

The posterior uncertainty ${\hat{\sigma }}_{Ci|R_{i,n}}^{2}$ is:

$${\hat{\sigma }}_{Ci|R_{i,n}}^{2}=\sigma_{Ci}^{2}-\frac{\sigma_{Ci}^{2}}{\sigma_{Ci}^{2}+\sigma_{Ri}^{2}}\sigma_{Ci}^{2}$$ (7)

The crucial proposal we advance is that the incentive value *V*_*i*_(*R*_*i*,*n*_)–attributed to a reward amount *R*_*i*,*n*_ in relation to the attribute *i* and associated with option *u*_*n*_–is central to belief updating (see Eq 6) and corresponds to a *precision-weighted prediction error* [49]; namely, to the difference between *R*_*i*,*n*_ and the prior mean μ_Ci,_ multiplied by a gain term which depends on the uncertainty of that attribute $\sigma_{Ri}^{2}$ and the prior uncertainty $\sigma_{Ci}^{2}$:

$$\begin{array}{l} V_{i}(R_{i,n})=\frac{\sigma_{Ci}^{2}}{\sigma_{Ci}^{2}+\sigma_{Ri}^{2}}(R_{i,n}-\mu_{Ci})\\ \overset{}{\Rightarrow }\\ {\hat{\mu }}_{Ci|R_{i,n}}=\mu_{Ci}+V_{i}(R_{i,n}) \end{array}$$ (8)

Within BCV, incentive value imbues reward and associated options with behavioural relevance, by favouring either approach to (for positive incentive values) or avoidance of (for negative incentive values) these reward amounts and options.

This implies two fundamental forms of contextual normalization. First, a subtractive normalization is exerted when *μ*_*Ci*_ is different from zero. For example, if we assign positive and negative numbers to rewards (i.e., *R*_*i*,*n*_ > 0) and punishments (i.e., *R*_*i*,*n*_ < 0) respectively, their corresponding incentive values will change in sign, depending on whether punishment (i.e., *μ*_*Ci*_ < 0) or reward (i.e., *μ*_*Ci*_ > 0) is expected a priori. Small rewards may appear as losses in contexts where large rewards are expected. Second, a divisive normalization depends on considering the gain term $\frac{\sigma_{Ci}^{2}}{\sigma_{Ci}^{2}+\sigma_{Ri}^{2}}$. This implies that the positive and negative value of profits (i.e., *R*_*i*,*n*_ > *μ*_*Ci*_) and losses (i.e., *R*_*i*,*n*_ < *μ*_*Ci*_) are magnified by a large gain term, when we have precise beliefs about the average reward of the population.

Sequential Bayesian belief updating means that inference proceeds by considering one reward amount at a time. If *R*_*i*,*n*_ is considered at step *t+1* and ***R***_*i*,*t*_ is a set containing all reward amounts already seen up until step *t* for attribute *i*, then a posterior mean $\mu_{Ci|R_{i,t}{,R}_{i,n}}$ is obtained at step *t+1* equivalent to (Bishop, 2006):

$${\hat{\mu }}_{Ci|R_{i,t}{,R}_{i,n}}={\hat{\mu }}_{Ci|R_{i,t}}+\frac{{\hat{\sigma }}_{Ci|R_{i,t}}^{2}}{{\hat{\sigma }}_{Ci|R_{i,t}}^{2}+\sigma_{Ri}^{2}}(R_{i,n}-{\hat{\mu }}_{Ci|R_{i,t}})$$ (9)

Implying a value for the reward amount *R*_*i*,*n*_:

$$V_{i}(R_{i,n})=\frac{{\hat{\sigma }}_{Ci|R_{i,t}}^{2}}{{\hat{\sigma }}_{Ci|R_{i,t}}^{2}+\sigma_{Ri}^{2}}(R_{i,n}-{\hat{\mu }}_{Ci|R_{i,t}})$$ (10)

For each attribute *i*, incentive values are accumulated in memory until inference is completed (i.e., all reward amounts have been considered). We can assume that inference proceeds in sequence or in parallel across attributes; however, this has no impact on incentive values, as the agent believes that attributes are associated with independent reward populations (formally: *P*(*C*_1_,*C*_2_,…,*C*_*I*_|***R***) = *P*(*C*_1_|***R***_**1**_), *P*(*C*_2_|***R***_**2**_),…,*P*(*C*_*I*_|***R***_***I***_)).

When all attributes for an option *u*_*n*_ have been considered, we assume that the incentive value of the option corresponds to the sum of the incentive values of associated reward amounts:

$$V(u_{n})=\sum_{i=1}V_{i}(R_{i,n})$$ (11)

Inference proceeds until, for all attributes *i*, the posterior expectation about rewards ${\hat{\mu }}_{Ci|Ri}$ is evaluated and, at this point, a choice is realized following a softmax rule based on the incentive values of the available options [2].

In summary, BCV is based on the following assumptions:

1. Each attribute is associated with an average reward (which is a hidden variable).
2. Average rewards for different attributes are independent.
3. For each attribute, the rewards offered (or observed rewards) are treated as samples that depend on the average reward.
4. The observed rewards are used to invert the model and infer the average reward.
5. During inference, observed rewards are considered sequentially.
6. During inference, an incentive value is calculated for each observed reward that corresponds to a precision-weighted prediction error.
7. Incentive values are summed across observed rewards and attributes, and choice follows a softmax rule.

Below these assumptions are discussed in detail. Assumptions (i), (iii), and (iv) are implicit in adopting a Bayesian scheme. Assumption (vii) is based on a standard approach in which incentive values are summed and a softmax choice rule is adopted. Assumption (ii) captures the notion of multiple attributes, in other words it enables an agent to link rewards to their attributes. Assumption (vi) (sequential belief updating or evidence accumulation) reflects the real world constraint that people have to evaluate available options and rewards one by one. In other words, agents cannot magically and instantaneously assimilate all the options on offer–they have to accumulate evidence for the underlying payoffs by evaluating each in turn. This notion plays a central role since we will see that context effects emerge because a reward is contextualized by previous rewards encountered during inference. This underlies assumption (v) that associates incentive value with a precision-weighted prediction error–a central construct in Bayesian inference. Heuristically, this scheme implies that an option is more likely to be selected if it increases expectations of reward, and will be avoided if it decreases expectations. In other words, an option is more likely to be selected if it suggests the situation is better than indicated by options considered previously during belief updating.

Note that a Bayesian perspective may suggest that incentive value corresponds to a posterior belief–rather than a precision-weighted prediction error. As an example, this would imply that the value of the same dish will be perceived as ‘higher’ in a ‘better’ restaurant. However, empirical data are consistent with the opposite notion that (adopting the same example) the value of the same dish is perceived as lower in a better restaurant [6–14]. This evidence motivated our proposal that incentive value corresponds to a precision-weighted prediction error, and not to a posterior belief.

In sum, BCV provides a principled explanation for how Bayesian inference, assigning a key role to prior expectation and uncertainty, might underlie value computation and choice. The key role of uncertainty is reflected in the precision-weighting of prediction errors. The hypothesis we entertain here is that the mechanisms postulated by BCV may be general and explain multiple forms of context effects. We have previously applied BCV to explain between-choice context effects; namely, those elicited by options presented in the past [11]. Here, we explore the possibility of applying the same model to within-choice effects, which arise when multiple options are available. In what follows, we will consider single and multiattribute choices under this Bayesian formalism.

### Single attribute decisions

**Fig 3:**
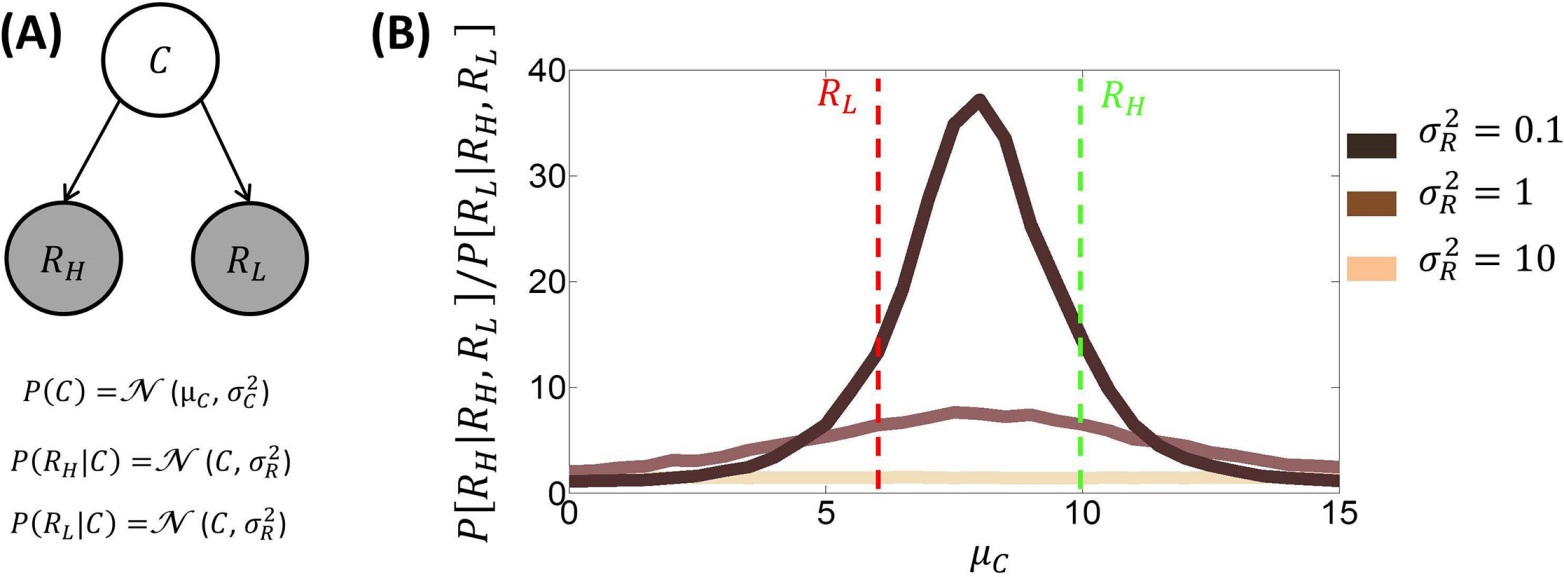
**A:** Generative model involved during choice between two options (characterized by a single attribute), one associated with high reward (*R*_*H*_ = 10) and the other with low reward (*R*_*L*_ = 6). **B:** Proportion of choices of the better over choices of the worse option predicted by BCV (*P*[*R*_*H*_|*R*_*H*_,*R*_*L*_]/*P*[*R*_*L*_|*R*_*H*_,*R*_*L*_], as a function of prior expectation μ_*C*_ and reward uncertainty $\sigma_{R}^{2}$ (100000 trials are simulated for each condition; $\sigma_{C}^{2}$ = 1 for simulations). BCV assumes a softmax choice rule (with inverse temperature parameter equal to one for all simulations) and an equal probability for each option of being considered during the first inference step. This shows bell-shape functions where peaks correspond to a prior mean expectation of (*R*_*H*_–*R*_*L*_)/2 and where the reward uncertainty $\sigma_{R}^{2}$ determines the width of the function (smaller uncertainties are connected with narrower functions).

Here, we apply BCV to explain within-choice contextual influences during non-multiattribute decisions. These comprise choices in which trading-off different attribute is not required, as for instance when options are defined by a single attribute. Consider first how different prior expectations *μ*_*C*_ (i.e., the prior expectation over the average reward of the attribute) and reward uncertainty $\sigma_{R}^{2}$ affect the choice between two options characterized by a single attribute (Fig 3). We can examine the predicted proportion of choosing a better option (associated with high reward *R*_*H*_) compared to a worse option (associated with low reward *R*_*L*_), as a function of prior expectation *μ*_*C*_ and reward uncertainty $\sigma_{R}^{2}$. Classical theories predict a flat function because they do not model an influence of the prior mean *μ*_*C*_ and reward uncertainty $\sigma_{R}^{2}$ [1–4]. In contrast, BCV predicts bell-shape functions over prior expectations, that peak at the prior mean of *μ*_*C*_ = (*R*_*H*_–*R*_*L*_)/2 (Fig 3). In this setting, the reward uncertainty $\sigma_{R}^{2}$ determines the width of the function (larger uncertainties produce narrower functions). Below, we analyse conditions where more than two options are available–and within-choice context effects come into play.

Classical decision-making models predict that, during choice, the choice ratio between two options should not be affected by the reward associated with a third option [1–4]. However, a recent study has challenged this hypothesis, highlighting within-choice context effects [6]. Adopting a choice task in which three options were available during choice, this study showed that the choice proportion between a more valuable and a less valuable target option diminished as a third option value increased towards the value of the target options (Fig 1A). After this point, the choice proportion started increasing (Fig 1A).

**Fig 4:**
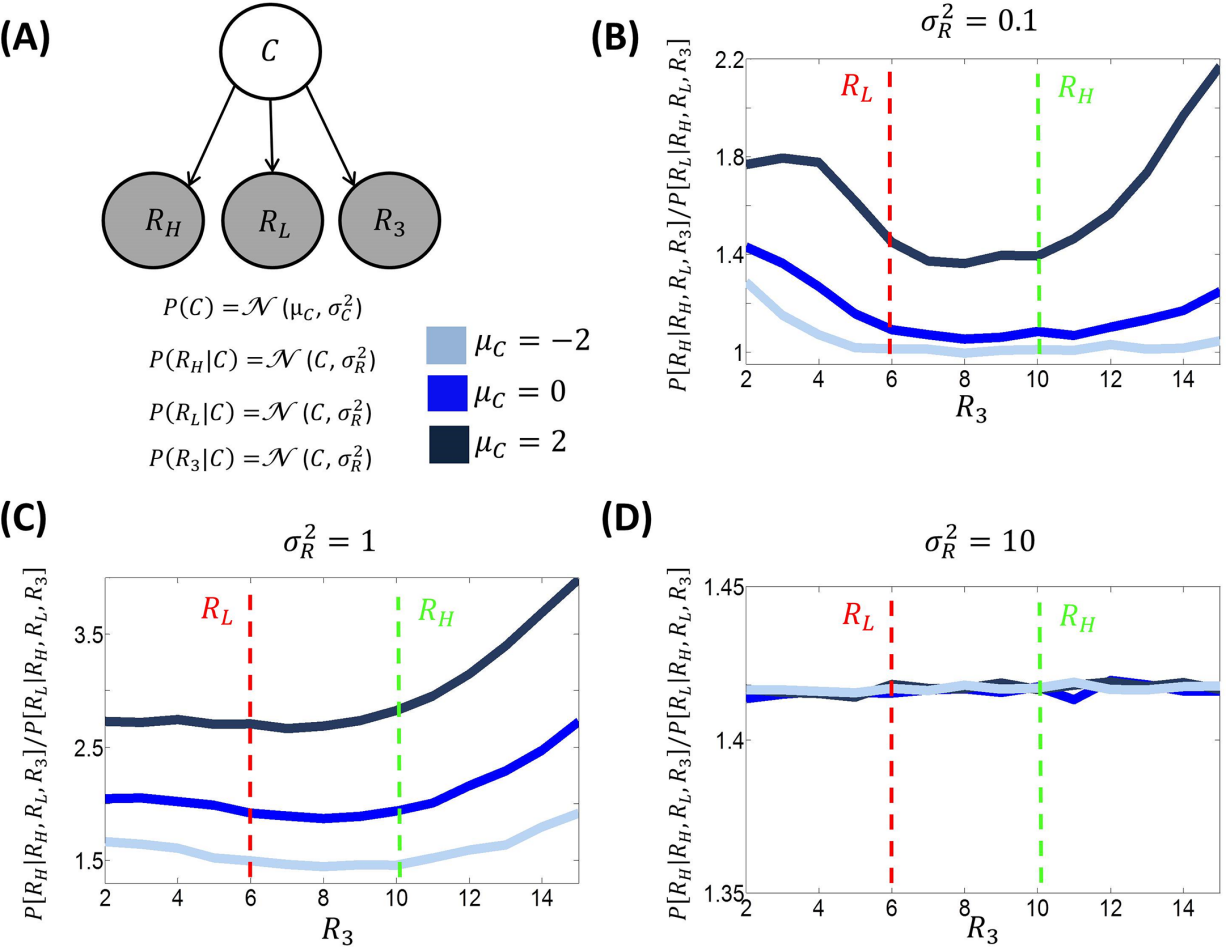
**A:** Generative model involved during choice between two options, one associated with high reward (*R*_*H*_ = 10) and the other with low reward (*R*_*L*_ = 6) when a third option (associated with reward *R*_3_) is also available [6]. **B:** Proportion of choices of the better over choices of the worse option predicted by BCV (*P*[*R*_*H*_|*R*_*H*_,*R*_*L*_,*R*_3_]/*P*[*R*_*L*_|*R*_*H*_,*R*_*L*_,*R*_3_]), as a function of the third option reward *R*_3_ and prior expectation μ_*C*_ (100000 trials are simulated for each condition; $\sigma_{C}^{2}$ = 1 for simulations). Here, the reward uncertainty $\sigma_{R}^{2}$ was set to 0.1; **C:** The same simulation is reported except that the reward uncertainty $\sigma_{R}^{2}$ was set to one. **D**: The same simulation is reported except that the reward uncertainty $\sigma_{R}^{2}$ was set to ten.

Here, we examine the implications of applying BCV in this scenario. Fig 4 illustrates the predictions of BCV of the ratio of choices of the two target options (a better target option *R*_*H*_ and a worse target option *R*_*L*_) as a function of the reward of a third option *R*_3_ and as a function of the agent’s prior belief about the average option reward *μ*_*C*_ and about the reward uncertainty $\sigma_{R}^{2}$. This figure shows that all these variables exert an influence. First, for certain values of reward uncertainty $\sigma_{R}^{2}$ and prior mean *μ*_*C*_, the reward of a third option *R*_3_ influences the choice proportion between the two target options according to a U-shape function, in a way that is consistent with empirical findings (Fig 1A). Second, the impact exerted by the reward of a third option *R*_3_ decreases as the reward uncertainty $\sigma_{R}^{2}$ increases. In other words, within-choice context effects emerge only with small reward uncertainty $\sigma_{R}^{2}$. This can be explained by the fact that a small $\sigma_{R}^{2}$ magnifies reward prediction error (RPE), enabling contextual effects to emerge. Third, when the reward uncertainty $\sigma_{R}^{2}$ is sufficiently small, the prior mean *μ*_*C*_ comes into play. Overall, a larger prior mean *μ*_*C*_ increases the choice proportion between the two target options (independently of the reward of a third option *R*_3_). Furthermore, the prior mean *μ*_*C*_ exerts a modulatory influence on the effect of the reward of a third option *R*_3_, as the effect exerted by *R*_3_ is enhanced with a larger prior mean *μ*_*C*_. Note that context effects exerted by *R*_3_ are obtained with *μ*_*C*_ = 0, which can be considered a default value for this parameter.

Collectively, these simulations provide proof of principle that BCV can explain within-choice contextual effects in single-attribute decisions that are remarkably similar to those seen in empirical studies [6]. In what follows, we now extend the explanatory scope of BCV to multiattribute problems.

### Multiattribute decisions

Empirical studies of multi-attribute decisions have highlighted three forms of effects, including the *similarity* [20–23], *attraction* [15, 24–27], and *compromise* effect [17, 25, 27]. Here, we apply BCV to multi-attribute decisions and ask whether the predictions that emerge from the model reproduce the context effects found empirically. To this aim, we consider two options (e.g., the two cars A and B described above) defined by two attributes (e.g., price *p* and quality *q*). Considering the reward amounts of car A, we assign *R*_*p*,*A*_ = 1 to price (low scores indicate high price) and *R*_*q*,*A*_ = 10 to quality. Conversely, when considering the reward amounts of car B, we assign *R*_*p*,*B*_ = 10 to price and *R*_*q*,*B*_ = 1 to quality. We now consider the choice probability difference between option A and option B as a function of the reward amounts *R*_*p*,*K*_ and *R*_*q*,*K*_ of a third option K.

**Fig 5:**
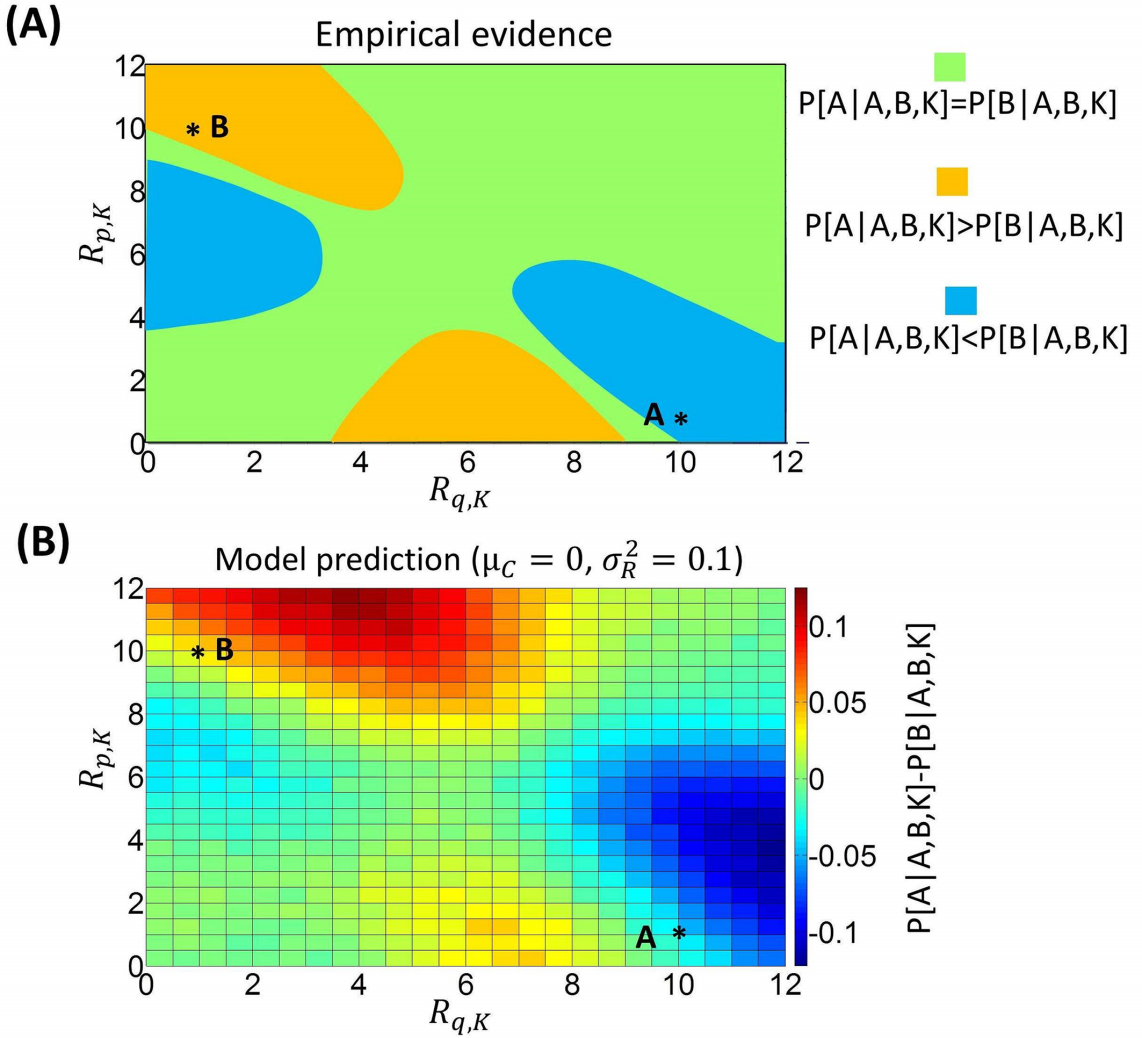
**A:** Empirical evidence (derived from integrating data from available studies as in [19]) concerning the difference in probability between choosing option A and option B when a third option K is available (*P*[*A*|*A*,*B*,*K*] − *P*[*B*|*A*,*B*,*K*]). Here options are characterized by two attributes (price *p* and quality *q*). For car A, we assign *R*_*p*,*A*_ = 1 to price (low scores indicate high price) and *R*_*q*,*A*_ = 10 to quality. For car B, we assign *R*_*p*,*B*_ = 10 to price and *R*_*q*,*B*_ = 1 to quality. The graph considers the choice probability difference between option A and option B as a function of the reward amounts *R*_*q*,*K*_ (for quality; x axis) and *R*_*p*,*K*_ (for price; y axis) of a third option K. Green areas indicate values for which no difference is expected based on empirical evidence; orange and blue areas indicates values for which a positive and negative difference is expected, respectively. **B:** The same analysis is performed with data simulated using BCV (100000 trials are simulated for each condition; μ_*C*_ = 0; $\sigma_{R}^{2}=0.1$; $\sigma_{C}^{2}$ = 1 for simulations).

Empirical evidence is hard to reconcile with standard models of choice, which predict that the choice probability difference between option A and option B should not depend on the value of a third option K. Fig 5A summarises the empirical findings by plotting the probability of choosing A minus the probability of choosing B as a function of the attributes of a third option K. This graph shows conditions in which the choice probability difference is bigger or smaller than zero, illustrating both a similarity and an attraction effect. Specifically, a similarity effect favours option A when option K is good in price and bad in quality (top-left of the graph), and favours option B when option K is bad in price and good in quality (bottom-right of the graph). An attraction effect favours option A when option K is bad in price and has an average quality (bottom-middle of the graph), and favours option B when option K has an average price and is bad in quality (middle-left of the graph).

We can now apply BCV to model choices in this scenario by analysing the influence on the choice probability (difference between option A and option B) of the prior mean *μ*_*C*_ (we use an equal prior mean for both attributes price and quality; formally: *μ*_*Cp*_ = *μ*_*Cq*_), the reward uncertainty $\sigma_{R}^{2}$ (we use an equal reward uncertainty for both attributes price and quality; formally: $\sigma_{Rp}^{2}=\sigma_{Rq}^{2}$), and the reward amounts *R*_*p*,*K*_ and *R*_*q*,*K*_, associated with price and quality respectively, of option K.

Fig 5B illustrates the choice probability difference (between option A and option B) with prior mean *μ*_*C*_ = 0 and reward uncertainty $\sigma_{R}^{2}=0.1$. Focusing on areas of the graph where a similarity effect can be tested (i.e., top-left and bottom-right), we see that the similarity effect is reproduced by BCV. Moreover, focusing on areas of the graphs where an attraction effect can be tested (i.e., bottom-middle and middle-left), we can see that this effect can also be explained by BCV. Collectively, these simulations provide proof of principle that, for some sets of values of the prior mean *μ*_*C*_ and of the reward uncertainty $\sigma_{R}^{2}$, BCV explains both a similarity and an attraction effect. Note that these effects are obtained with *μ*_*C*_ = 0, which can be considered a default value for this parameter.

**Fig 6:**
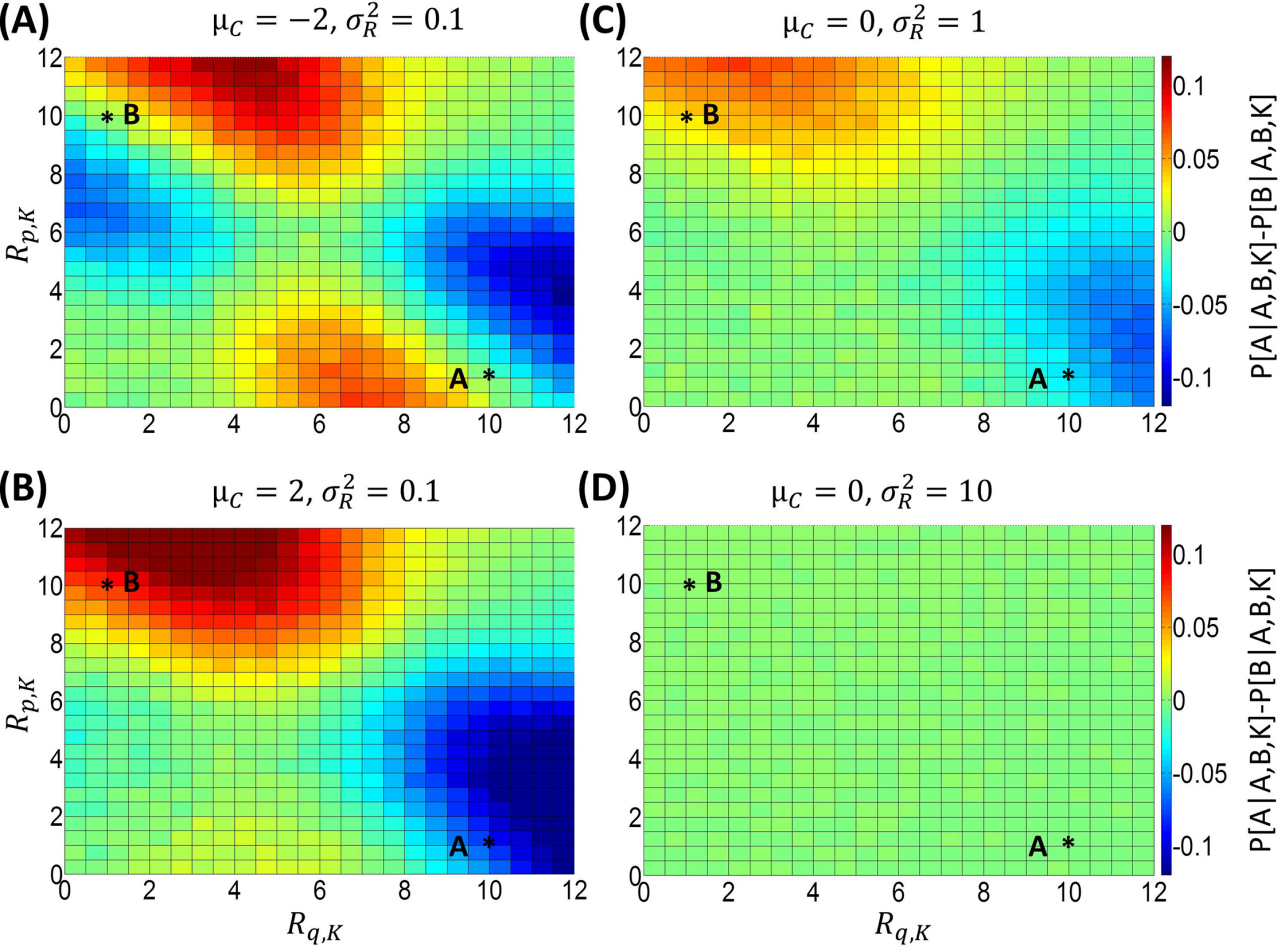
Predictions of BCV about the difference in probability between choosing option A and option B when a third option K is available (*P*[*A*|*A*,*B*,*K*] − *P*[*B*|*A*,*B*,*K*]). Here options are characterized by two attributes (price *p* and quality *q*). For car A, we assign *R*_*p*,*A*_ = 1 to price (low scores indicate high price) and *R*_*q*,*A*_ = 10 to quality. For car B, we assign *R*_*p*,*B*_ = 10 to price and *R*_*q*,*B*_ = 1 to quality. The graph considers the choice probability difference between option A and option B as a function of the reward amounts *R*_*q*,*K*_ (for quality; x axis) and *R*_*p*,*K*_ (for price; y axis) of a third option K (100000 trials are simulated for each condition; $\sigma_{C}^{2}$ = 1 for simulations). Different parameter sets are shown. **A:** Simulation using μ_*C*_ = −2 and $\sigma_{R}^{2}=0.1$. **B:** Simulation using μ_*C*_ = 2 and $\sigma_{R}^{2}=0.1$. **C:** Simulation using μ_*C*_ = 0 and $\sigma_{R}^{2}=1$. **D:** Simulation using μ_*C*_ = 0 and $\sigma_{R}^{2}=10$.

Fig 6A and 6B examines the effects of adopting other values of the prior mean *μ*_*C*_ (fixing the reward uncertainty $\sigma_{R}^{2}$ to 0.1) in this scenario. This figure shows that an attraction effect is obtained when the prior mean *μ*_*C*_ is smaller (*μ*_*C*_ = −2 in our simulation), but no similarity effect emerges. Conversely, a similarity effect is evident when the prior mean *μ*_*C*_ is larger (*μ*_*C*_ = 2 in our simulation), but the attraction effect vanishes.

Fig 6C and 6D illustrates the choice probability difference (between option A and option B) for different values of the reward uncertainty $\sigma_{R}^{2}$ (the prior mean *μ*_*C*_ was fixed to zero). We can see that both similarity and attraction effects are not detectable when reward uncertainty $\sigma_{R}^{2}$ is high. For smaller values of uncertainty, a similarity effect emerges but there is no attraction effect. Both effects can be obtained only when the reward uncertainty $\sigma_{R}^{2}$ is sufficiently low (Fig 5B). This highlights the role of the reward uncertainty $\sigma_{R}^{2}$ in determining the degree of contextual effects.

In summary, our analyses show that, when simulating multi-attribute decisions with BCV, similarity and attraction effects emerge for appropriate values of the prior mean *μ*_*C*_ and the reward uncertainty $\sigma_{R}^{2}$. The first parameter regulates the balance of the two effects, as an attraction effect (but no similarity effect) is obtained when the prior mean *μ*_*C*_ is small, while a similarity effect (but no attraction effect) is obtained when the prior mean *μ*_*C*_ is large. Both effects emerge for intermediate values of the prior mean *μ*_*C*_, including a prior mean *μ*_*C*_ = 0, which is a default value for this parameter. The reward uncertainty $\sigma_{R}^{2}$ plays a key role too, because context effects vanish when this parameter is high. Decreasing levels of reward uncertainty $\sigma_{R}^{2}$ reveal a similarity effect first and then an attraction effect. These results indicate that the similarity and attraction effects arise naturally from BCV, without any *ad-hoc* assumptions–and under natural values of model parameters (prior mean *μ*_*C*_ reward uncertainty $\sigma_{R}^{2}$).

A compromise effect [17, 25, 27] has been observed when the choice set includes two options scoring high in one attribute and low in another, in addition to a third option with intermediate scores for both attributes. Crucially, the three options are equally preferred (i.e., are chosen an equal amount of times) if presented in pairs during binary choices. However, when they are available altogether, a preference for the option characterized by intermediate scores is seen. We model this scenario by manipulating the distance between attributes for two options A and B, namely assigning *R*_*p*,*A*_ = 5 − *d* and *R*_*q*,*A*_ = 5 + *d* for option A, and *R*_*p*,*B*_ = 5 + *d* and *R*_*q*,*B*_ = 5 − *d* for option B, where the *proximity parameter d* varies (across simulations) from zero to four. To represent the option with intermediate scores for both the two attributes, we assign *R*_*p*,*K*_ = 5 and *R*_*q*,*K*_ = 5.

**Fig 7:**
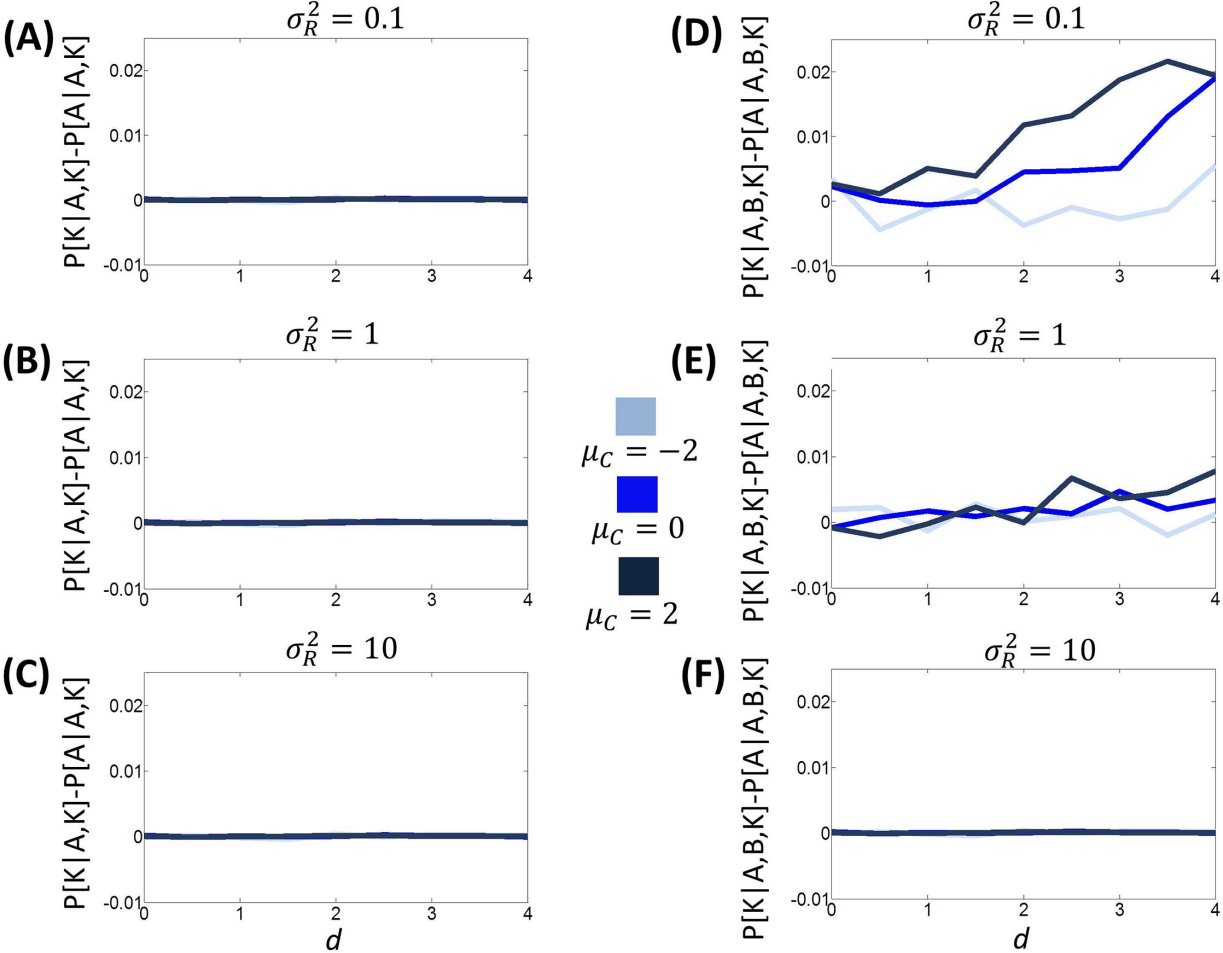
Predictions of BCV related to the compromise effect, involving option A, B and K. Here, in relation with option A, we assign *R*_*p*,*A*_ = 5 − *d* for price and *R*_*q*,*A*_ = 5 + *d* for quality; in relation with option B, we assign *R*_*p*,*B*_ = 5 + *d* for price and *R*_*q*,*B*_ = 5 − *d* for quality. The proximity parameter *d* varies across simulations from zero to four. To represent the option K with intermediate scores for both the two attributes, we assign *R*_*p*,*K*_ = 5 and *R*_*q*,*K*_ = 5 (100000 trials are simulated for each condition; $\sigma_{C}^{2}$ = 1 for simulations). **A**: The difference in probability between choosing option K and option A during binary choice (*P*[*K*|*A*,*K*] − *P*[*A*|*A*,*K*]). The reward uncertainty $\sigma_{R}^{2}$ is set to 0.1 and different values of the prior mean μ_*C*_ are considered. **B:** The same simulation is reported except that the reward uncertainty $\sigma_{R}^{2}$ was set to one. **C:** The same simulation is reported except that the reward uncertainty $\sigma_{R}^{2}$ was set to ten. **D**: The difference in probability between choosing option K and option A during choices in which option B is also available (*P*[*K*|*A*,*B*,*K*] − *P*[*A*|*A*,*B*,*K*]). The reward uncertainty $\sigma_{R}^{2}$ is set to 0.1 and different values of the prior mean μ_*C*_ are considered. **E:** The same simulation is reported except that the reward uncertainty $\sigma_{R}^{2}$ was set to one. **F:** The same simulation is reported except that the reward uncertainty $\sigma_{R}^{2}$ was set to ten.

Fig 7A, 7B and 7C shows the prediction of BCV using these settings during binary choices between option K and option A, using different parameters for the prior mean *μ*_*C*_ and the reward uncertainty $\sigma_{R}^{2}$. The results indicate that the choice probability difference is always zero, irrespective of the values of the proximity parameter *d* or the parameters of the model (prior mean *μ*_*C*_ and reward uncertainty $\sigma_{R}^{2}$). Fig 7D, 7E and 7F shows the choice probability difference between option K and option A, when option B is also available. For certain values of the parameters (prior mean *μ*_*C*_ and reward uncertainty $\sigma_{R}^{2}$), this difference is zero with *d* = 0 and increases with the proximity parameter *d*. This effect disappears when reward uncertainty $\sigma_{R}^{2}$ is too large or when the prior mean *μ*_*C*_ is too small. Overall, these results show that the compromise effect emerges naturally from BCV, without any *ad-hoc* assumptions and under default values of the parameters (prior mean *μ*_*C*_ and reward uncertainty $\sigma_{R}^{2}$). Interestingly, these simulations predict a correlation between the compromise effect and the proximity parameter *d*, reflecting differences between the intermediate and extreme options. This phenomenon is predicted by another model of the compromise effect [19] but remains to be validated empirically.

In summary, these simulations provide proof of principle that BCV predicts within-choice contextual effects during multiattribute decisions that are remarkably similar to those seen in empirical studies. In other words, the similarity, attraction and compromise effects seen empirically are all emergent properties of BCV. In the next section, we turn from within choice effects and consider between-choice context effects.

### Between-choice context effects

**Fig 8:**
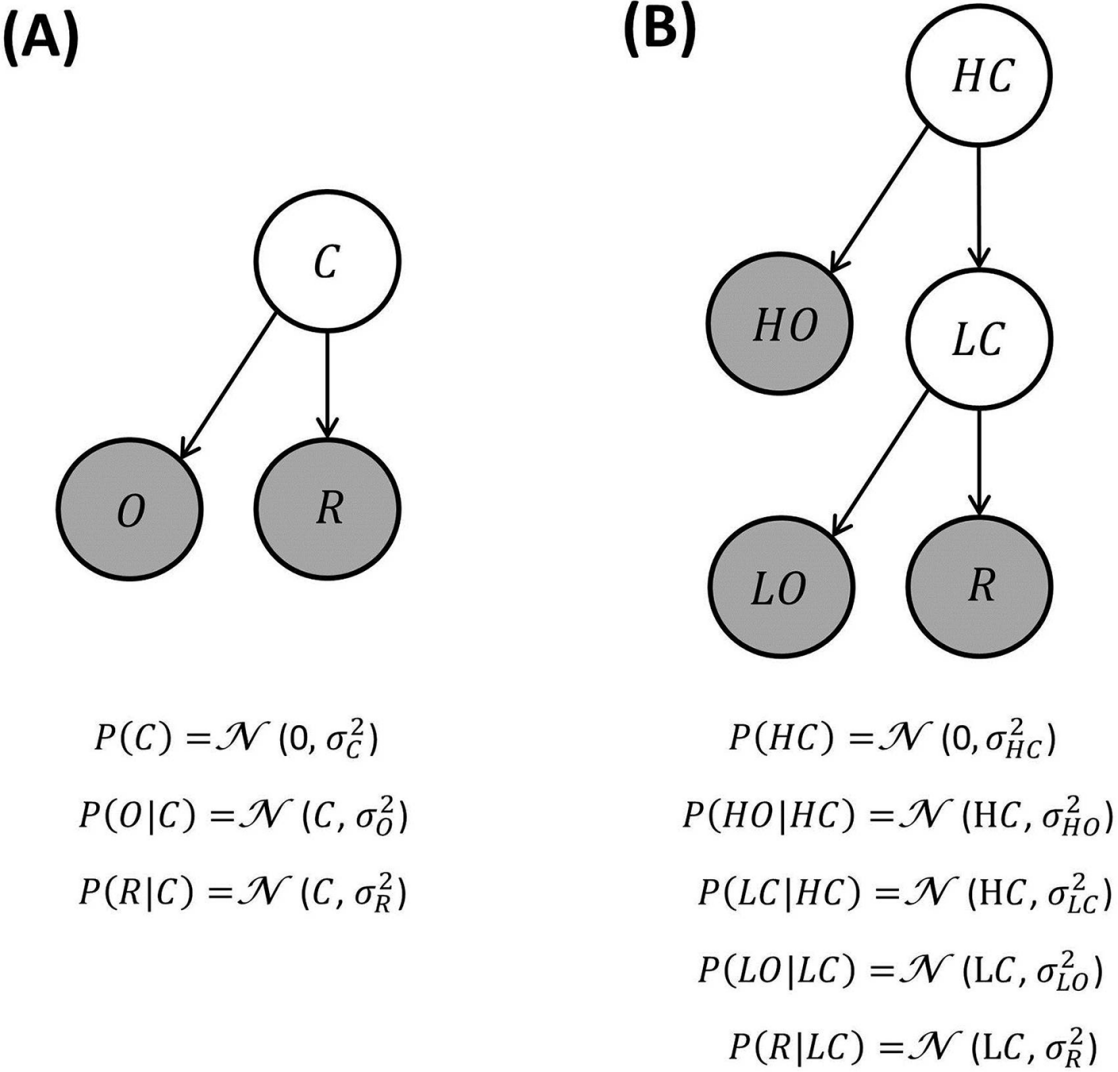
**A:** Generative model where a contextual variable C reflects a prior expectancy of zero over the reward mean, and a noisy observation O of the context value is provided. **B:** Generative model where context is organized hierarchically and comprises a high level (HC; e.g., a neighbourhood) and a low level (LC; e.g., a restaurant), both associated with noisy observations (HO and LO respectively).

To characterize between-choice context-effects [11], BCV uses the same generative model as above, characterized by a prior belief *μ*_*C*_ (here we consider only options defined by a single attribute) over reward (with uncertainty $\sigma_{C}^{2}$) and by an observation of reward amount *R* (with uncertainty $\sigma_{R}^{2}$). Here, the generative model is extended to include a Gaussian observation variable O that reflects contextual information provided before an option is presented (Fig 8A). This depends on the hidden cause *C* and is endowed with uncertainty $\sigma_{O}^{2}$ (as for the reward amount):

$$O∼N(C,\sigma_{O}^{2})$$ (12)

As above, we assume that an agent infers the posterior expected reward of options afforded by a given context, based on the reward amount but also now on contextual information (i.e., ${\hat{\mu }}_{C|O,R}$). Since the latter is provided before the option, we assume that the agent infers ${\hat{\mu }}_{C|0}$ first and then ${\hat{\mu }}_{C|O,R}$, when the option is presented. Assuming a prior mean equal to zero *μ*_*C*_ = 0, then:

$${\hat{\mu }}_{C|0}=\frac{\sigma_{C}^{2}}{\sigma_{C}^{2}+\sigma_{O}^{2}}O$$ (13)

And the posterior uncertainty:

$${\hat{\sigma }}_{C|O}^{2}=\sigma_{C}^{2}-\frac{\sigma_{C}^{2}}{\sigma_{C}^{2}+\sigma_{O}^{2}}\sigma_{C}^{2}$$ (14)

The mean of the posterior distribution P(C|O,R) corresponds to:

$${\hat{\mu }}_{C|O,R}={\hat{\mu }}_{C|0}+\frac{{\hat{\sigma }}_{C|O}^{2}}{{\hat{\sigma }}_{C|O}^{2}+\sigma_{R}^{2}}(R-{\hat{\mu }}_{C|0})$$ (15)

Implying the following incentive value for the option:

$$V(R)=\frac{{\hat{\sigma }}_{C|O}^{2}}{{\hat{\sigma }}_{C|O}^{2}+{\hat{\sigma }}_{R}^{2}}(R-{\hat{\mu }}_{C|0})$$ (16)

This shows that, other things being equal, information about context (reflected in the value of O) induces subtractive value normalization. For instance, when contextual cues O supports a larger reward, ${\hat{\mu }}_{C|0}$ will be larger and hence the reward prediction error (i.e., $R-{\hat{\mu }}_{C|0}$) will be smaller.

An extension of this generative model is illustrated in Fig 8B, where contexts are organized hierarchically. Combining the influence of reward expectancies within a hierarchy allows the generative model to explain the impact of context at multiple levels. For instance, the value attributed to a certain dish may depend on the reward distribution associated with a restaurant (a more specific context), integrated with the reward distribution associated with a city (a more general context). In detail, a higher-level prior belief about the average reward amount of options (e.g., at the level of the neighbourhood) is represented by a Gaussian distribution with mean *μ*_*HC*_ equal to zero and uncertainty $\sigma_{HC}^{2}$, from which a value HC is sampled. Contextual information about HC is provided and represented by HO that is sampled from a Gaussian distribution with mean HC and uncertainty $\sigma_{HO}^{2}$. A lower-level belief about the average reward amount of options (e.g., the restaurant) is represented by a (Gaussian) distribution with mean HC and uncertainty $\sigma_{LC}^{2}$, from which a value LC is sampled. Contextual information about LC is provided and represented by LO, which is sampled from a Gaussian distribution with mean LC and uncertainty $\sigma_{LO}^{2}$. A reward is obtained and sampled from a Gaussian distribution with mean LC and uncertainty $\sigma_{R}^{2}$.

We propose that agents infer the posterior expectation ${\hat{\mu }}_{LC|HO,LO,R}$ P(LC|HO,LO,R) sequentially by estimating ${\hat{\mu }}_{HC|HO}$, ${\hat{\mu }}_{LC|HO}$, ${\hat{\mu }}_{HC|HO,LO}$ and finally ${\hat{\mu }}_{LC|HO,LO,R}$. This produces an equation for incentive value with the following form (see Materials and Methods for derivation):

$$V(R)=K(R-\tau_{LO}LO-\tau_{HO}HO)$$ (17)

Three normalization factors are implicit here. The first (*τ*_*LO*_*LO*) is a subtractive normalization factor proportional to the value LO observed at the low contextual level. The second (*τ*_*HO*_*HO*) is a subtractive normalization factor proportional to the value HO observed at the high contextual level. The terms τ represent gain-dependent effects and describe the relative precision of information conveyed by the low-level (*τ*_*LO*_) and high-level (*τ*_*HO*_) observations. Finally, a third factor (K) implements divisive normalization and depends on a gain term which includes reward uncertainty (see Materials and Methods for details).

In recent studies [8–11], we have investigated the nature of contextual influence on incentive value that depends on reward expectations established before choice presentation (between-choice effects). In these studies, we have used a simple decision-making task, where participants had to repeatedly choose between a sure monetary reward and a fifty-fifty gamble. These options comprised double the sure monetary reward and a zero outcome, ensuring that the two options had equivalent expected reward or value (EV). Across blocks, we manipulated the distribution of EVs, such that these distributions overlapped. We analysed choice behaviour with EVs common to both contexts to examine whether incentive value attributed to the objective EV changed according to BCV predictions.

**Fig 9:**
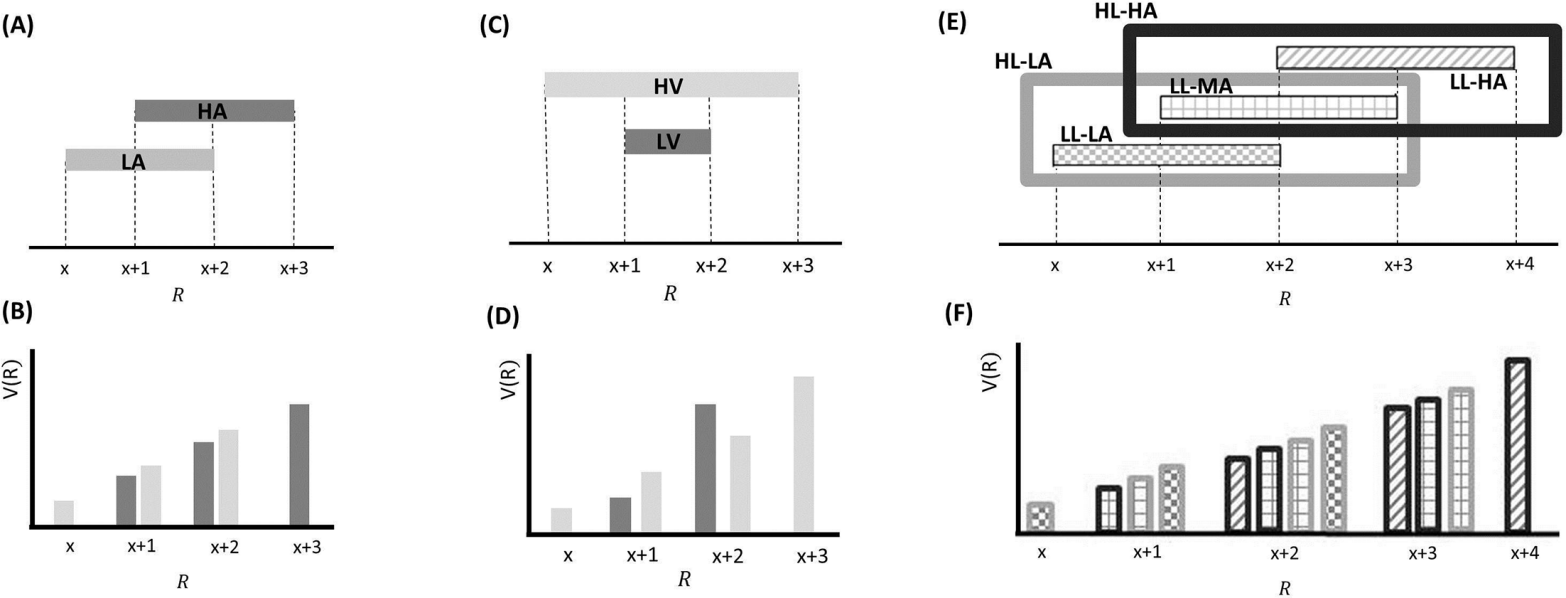
Between-choice effects (predicted by BCV) that depend on contexts with different averages. Contexts are associated with certain distribution of rewards presented sequentially over trials (arranged in blocks for each context). **A:** Example with a single hierarchical level, where two contexts have different average rewards. In blocks associated with a low-average context (LA; in lighter grey), possible rewards are x, x+1 and x+2; in blocks associated with a high-average context (HA; in darker grey), possible rewards are x+1, x+2 and x+3. **B:** BCV prediction of the incentive value attributed to rewards depending on these contexts. Larger values are predicted in the LA compared to the HA for amounts common to both contexts. **C:** Effects predicted by BCV dependent on contexts with different variance. In blocks associated with a high-variance context (HV; in lighter grey), possible rewards are x, x+1 x+2 and x+3; in blocks associated with a low-variance context (LV; in darker grey), possible rewards are x+1 and x+2. **D:** BCV prediction of the incentive value attributed to rewards depending on these contexts. Considering rewards common to both contexts, BCV predicts higher incentive value for x+1 in the high-variance context and for x+2 in the low-variance context. **E:** Example with two hierarchical levels (low-level (LL) contexts, represented by filled rectangles, and high-level (HL) contexts, represented by frames). Blocks associated with HL contexts comprise several sub-blocks associated with LL contexts having specific average reward. In the HL context with low-value (HL-LA; light frame), a LL context with low average (LL-LA, where rewards are x, x+1 and x+2) and a LL context with medium average (LL-MA, where rewards are x+1, x+2 and x+3) alternate. In the HL context with high-value (HL-HA; dark frame), a LL-MA context and a LL context with high average (LL-HA, where rewards are x+2, x+3 and x+4) alternate. **F:** BCV prediction of the incentive value attributed to rewards depending on these contexts. The fill colour of bars represent the LL context condition, the outline colour represent the HL context condition. BCV predicts that incentive values derive from integrating both hierarchical levels, with larger values emerging when average reward is lower at both context levels.

In one experiment (Fig 9A and 9B; [8, 9]), in different blocks, the sure monetary gain was drawn from one of two distinct, but partially overlapping, distributions of rewards (low-average and high-average context). Choice behaviour was consistent with attributing a larger incentive value to common EVs in the low average compared to high-average context. This and similar evidence [5–14, 50] suggests that incentive values are, to some extent, rescaled to the average reward expected in a given context, such that they increase (resp. decrease) with smaller (resp. larger) average reward expectations.

These data fit within predictions of BCV. In addition, BCV postulates a between-choice influence of expected reward variance on incentive values (Fig 9C and 9D). In a recent study [11], we used the same gambling task described above and manipulated contextual variance on two levels; one associated with blocks where two target trial EVs were presented (low-variance context), and another with blocks where the same two target trial EVs plus a larger and a smaller EV were presented (high-variance context). Crucially, this ensured that the two contexts had equivalent average reward but different variance. BCV predicts that the incentive value of the smaller target trial EV will be lower in the low-variance compared to the high-variance context, and the incentive value of the larger target trial EV will be higher in the low-variance compared to the high-variance context. In other words, BCV predicts a larger value difference between the two target trial EV in the low compared to high-variance context. This derives from the gain term, which depends on contextual reward variance. Specifically, low variance magnifies the reward prediction error and hence further reduces the value of rewards that are lower than expected and enhances the value of rewards that are larger than expected. We have previously provided data that are consistent with this prediction [11].

This latter study supports the hypothesis that between-choice reward variance influences incentive value consistent with BCV. In the same study (Fig 9E and 9F; [11]), we also reported that between-choice context effects can be expressed at different hierarchical levels, in line with predictions of BCV. Participants played a computer-based task, where two decks of cards (representing a low-level context) appeared. Each card was associated with a monetary reward, and decks contained cards with different average rewards. A card was drawn from a selected deck and participants had to choose between half of the card reward for sure and a gamble between the full reward and a zero outcome, each with 50% chance. Two sets of decks (representing a high-level context) alternated in a pseudo-random way. The empirical data showed that the lowest incentive values were attributed when both high-value decks and deck-sets were simultaneously presented, while the highest incentive values were attributed when low-value decks and deck-sets were simultaneously presented. Intermediate incentive values were attributed when decks and deck-sets had one high value and the other low value.

Collectively, these empirical studies provide evidence consistent with between-choice contextual effects on incentive value that depends on beliefs about the average reward and variance expected across choices at multiple hierarchical levels. Furthermore, the empirical findings endorse the predictions derived from BCV.

## Discussion

We advance BCV as a unifying theory of contextual effects in value-based choice under the normative principles of Bayesian statistics. BCV assumes that the brain calls on Bayesian inference to invert a generative model and compute (independently for each attribute) the average reward based on observing different reward amounts of options that are available in a given context. Our key proposal is that incentive value emerges during this inferential process, and corresponds to a precision-weighted reward prediction error. Here, we show that these principles are sufficient to explain a wide range of between-choice and within-choice contextual influences; in the latter case encompassing both single and multiattribute effects. To our knowledge, this is the first time a theory has been applied to the full range of context effects.

An important advantage of BCV is its grounding in normative principles of Bayesian statistics [32–37]. Several arguments have been made in support of a Bayesian approach. These are based on a formal and clear definition of the functions that motivate cognitive processes, which are formulated as Bayesian inference and learning. This allows BCV to establish a direct link with Bayesian schemes in other domains–a step towards formulating a unifying theory of brain function. Remarkably, we show that the same basic processes postulated by BCV can be applied to a wide range of conditions in which contextual effects on value and choice are involved. Beyond explaining the available empirical evidence, this scheme can generate new hypotheses (see below). Indeed one of our previous studies [11] was motivated by testing predictions arising out of our initial formulations of BCV.

BCV is associated with planning as inference and active inference [40–45]. The basic idea is that an agent considers the rewards on offer as samples drawn from a population. The latter is not known directly, but can be inferred based on the rewards on offer. Heuristically, agents are interested in inferring how much reward is available on a given trial, which they estimate by combining prior expectations with observations of available rewards. On this view, agents primarily aim to *infer*–and not maximize–the reward; implying that utility-maximization is an emergent process. We argue that an advantage of this perspective is that it offers a normative interpretation of contextual effects, which emerge from the inferential treatment offered here.

Although our theoretical treatment is grounded in Bayesian inference one might argue that the Bayesian gloss is unnecessary to understand the particular inferential mechanisms we have called upon [51]. To a certain extent, there is tautology in Bayesian explanations for behaviour. This follows from the complete class theorem (i.e., for every loss function and behaviour there is a prior belief that renders the behaviour Bayes optimal) [52]. In other words, in principle, everything is Bayes optimal under some priors. This means that the interesting questions reduce to the form of prior beliefs that constitute a subject’s generative model. Our focus has been on the form of these models and the particular role of precision weighting in belief updating and choice. The results of our analysis are consistent with empirical data on several forms of context effect, and hence may contribute to a clarification of the computational principles at play.

In BCV incentive value, and in turn choice behaviour, emerges from Bayesian belief updating. Under continuous state space models of the hidden causes of reward values, belief updates and incentive value can be cast as precision-weighted (reward) prediction error. A possibility consistent with BCV is that action is steered by (precision-weighted) prediction errors and is oriented to error cancellation, with approach and avoidance responses elicited by positive and negative prediction errors, respectively. The crucial role of prediction error highlights a perspective in which incentive value is inherently *relative* with respect to reward expectation. Eliciting approach and avoidance behaviour in response to positive and negative prediction errors can be conceived as a basic error-cancellation process (crystallized during evolution of biological organisms), which is a core tenet of active inference schemes.

BCV postulates that the two fundamental determinants of incentive value are prediction error and relative precision. A prediction error is determined by the difference between the observed and expected reward which, in BCV, derives from integrating different expectations under contextual uncertainty. Relative precision depends on the (relative) precision or prior confidence–and ensures that the prediction error is normalised and (Bayes) optimally weighted in relation to uncertainty about both context and reward cues. BCV predicts precision exerts an influence in two ways. First, at high hierarchical levels, precision determines the optimal integration of multiple contextual representations–as it mandates that contexts characterized by a high precision (greater reliability) will exert more influence on reward expectancy. For instance, if we assume that subjects have very precise beliefs about the low-level context (e.g., the card deck in the final experiment on between-choice context effects), then the effect of the high-level (e.g., the deck set) will disappear. Formally, this is because in the hierarchical model the low-level context constitutes a Markov blanket for the posterior expectation about the reward option (Bishop, 2006). In other words, the effect of the high-level context tells us that if subjects are using a hierarchical model, there must be posterior uncertainty about the low-level context. Heuristically, even though they can see which deck they are currently playing with, they still nuance their expectations about this deck based upon the deck-set from which it came. Second, at the lowest hierarchical level, precision determines the gain assigned to the prediction error and hence is a direct determinant of incentive value.

Within BCV, the ratio between reward uncertainty and prior uncertainty determines the gain term (or relative precision) which is used for belief updating (see Eq 6). This means that manipulating the prior uncertainty produces exactly opposite effects compared to manipulating the reward uncertainty, meaning that varying one during simulations is sufficient for testing the predictions of the model (above, we manipulated reward uncertainty and kept the prior uncertainty constant). Thus BCV has only two parameters; namely, the prior mean and reward uncertainty. The role of the latter is straightforward, as context effects are allowed only with small reward uncertainty, and the size of these effects decreases with this reward uncertainty. The role of the prior mean is more complex: for instance, a large prior mean permits a similarity effect but interferes with an attraction effect, while a small prior mean allows an attraction effect but interferes with a similarity effect. Notably, all contextual effects are expressed when setting the prior reward expectation to zero, which can be considered the default value. In short, relying on only two parameters endows BCV with simplicity and constrains the predictions that can be derived, making BCV easy to validate or falsify (see below).

### Comparison with other models

We have shown that the principles underlying BCV can explain a wide range of empirical findings on the context sensitivity of value-based choice. Several previous accounts have focused on a single context effect, especially during multiattribute decisions. Some models have been developed explicitly for explaining the similarity effect [20, 53–55], other models for explaining the attraction effect [56, 57], and other models for the compromise effect [27]. However, a shortcoming of these models is their inability to explain all three effects within a single formal framework. More recently, adopting connectionist architectures, the multi-alternative decision field theory [16, 58, 59] and the leaky competing accumulator [19, 59, 60] have been able to reproduce all three effects (see also [61]). The first model [16, 58] is based on a process modelling attentional switches across attributes and a comparator mechanism which, for the attribute under attention, computes the difference between the reward of each option and the mean reward across options. The second model [19, 59, 60] is similar, except that the comparator applies a non-linear asymmetric (loss-averse) value function to the difference. Although these models fit remarkably with empirical literature and shed light on the neural mechanisms underlying choice, we argue that BCV presents several advantages. First, it is based on normative principles of Bayesian inference. This constrains the model in terms of empirical predictions. In other words, the similarity, attraction and compromise effect are implicit in the way the model works. In fact, these effects arise when defaults parameters are used. Second, BCV is a more parsimonious model; as the number of free parameters is much lower (essentially, the prior mean and the reward uncertainty). Third, without any further assumptions, BCV applies to a wider range of phenomena including single-attribute decisions and also accounts for between-context effects. Overall, while previous connectionist models are informative especially at the implementation level, BCV helps clarify context sensitivity at the algorithmic and computational level.

The concept of *wealth* in expected utility theory [3] and *status quo* in prospect theory [62] have been recently re-casted in terms of average expected reward [29]. This formulation opens the possibility of context effects dependent on changes in reward expectation. In line with this view, empirical evidence indicates a between-choice context effect that depends on the average contextual reward (as for example inferred from past choices), consisting in attributing larger incentive values in contexts characterized by lower reward. A similar idea has inspired decision by sampling theory [14, 31], which evokes a few basic cognitive processes to explain choice behaviour. According to this model, each choice option elicits retrieval from memory (in the form of random sampling) of stimuli encountered in the past, especially those associated with the current context. A set of binary comparisons follows between the option and the samples, and the number of comparisons in which the option is favoured over each sample is recorded. This number corresponds to the incentive value of the option and is computed for all options available, hence determining their relative preference. Since samples are drawn from memory, they depend on past experience and therefore reflect the distribution of options and outcomes characterizing the environment of an agent. This model can account for an attribution of larger incentive value to the same reward in contexts where lower compared to higher reward is expected before options are provided. This effect is explained by a decreased likelihood, in the former compared to the latter context, of sampling stimuli from memory that are preferred to rewards common to both contexts (assuming a recency effect in memory sampling; [14, 31]. BCV extends these views by appealing explicitly to Bayesian principles (i.e. Bayesian belief updating and evidence accumulation), with implications for empirical predictions. For instance, contrary to BCV and empirical findings, it remains unclear whether these previous models can account for between-choice contextual influence of reward variance or any within-choice contextual effects.

Divisive normalization theory [6, 63–68] has been proposed recently to explain both between-choice and within-choice contextual effects during single attribute decisions. Divisive normalisation was first proposed in the sensory domain to explain phenomena such as neural adaptation within the retina to stimuli of varying intensity [63]. There is evidence that similar principles can explain higher-order cognitive processes, such as selective attention and perceptual decision-making [63, 69]. Recently, divisive normalisation has been extended to contextual adaptation effects in value-guided choice [6], and proposes that incentive value corresponds to the reward divided by the average reward of past or current choices. This can explain contextual influences elicited both within-choice effects during non-multiattribute decisions and between-choice effects that depend on the average contextual reward. Though this scheme relies on a normalization scheme similar to BCV, different empirical predictions arise. It remains unclear whether this divisive normalization scheme is able to explain between-choice effects deriving from reward variance, and can explain data on multi-attribute choices. In addition, BCV, but not divisive normalization theory, is based on normative principles of Bayesian statistics. However, an attractive aspect of divisive normalization theory is the explicit connection with mechanisms characterizing biological neural processes [63]. A similar connection can be motivated for BCV, given several proposals showing how Bayesian inference (the framework of BCV) is compatible with neuronal processes [49, 70, 71].

The manner in which BCV conceptualizes incentive value is similar to recent economic models that postulate incentive value is adapted to the statistics of the expected reward distribution [29, 30]. These theories can be broadly classified into those based on subtractive normalization, which assume that incentive value corresponds to the reward minus a reference value [29], and those based on divisive normalization, assuming that incentive value corresponds to the reward divided (or multiplied) by the range of an expected distribution of rewards [30]. An important difference between BCV and these theories is the derivation of the former but not the latter from normative assumptions of Bayesian inference. From Bayesian belief updating, BCV derives the proposition that incentive value corresponds to precision-weighted prediction error, hence implying both a subtractive normalization to the expected reward and a divisive normalization with respect to the reward uncertainty. Importantly, these predictions are not *ad hoc* but derive from Bayesian assumptions, distinguish BCV from other models, and have been recently supported empirically [11]. In addition, while these recent economic models focus on between-choice context effects, BCV is more general as it can reproduce within-choice effects in both single and multiattribute decisions.

Like BCV, a recent proposal has interpreted multi-attribute within-choice effects based on the notion that perception of reward is stochastic [72]. The idea is that, for each attribute, an agent forms noisy observations of reward amounts and of the ordinal positions of the reward amounts. Multi-attribute effects can then be obtained by integrating these two observations [72]. Though there are analogies between BCV and the model of Howes et al. [72], we emphasize several important differences. First, the latter does not employ a Bayesian framework, since it is not based on integrating prior beliefs and observations, nor it is based on optimal weighting of different sources of information (as in multi-sensory integration). Second, the model of Howes et al. [72] has been applied to aspects of multi-attribute effects (such as the impact on reaction times), which remain to be explored with BCV. On the other hand, the model of Howes et al., [72] remains to be explored in relation to within-choice effects involving a single attribute and in relation to between-choice effects.

### Predictions and limitations of BCV

Specific empirical predictions can be derived from BCV, and here we highlight some of these. Standard economic theories assume that choice should be independent of whether options are presented simultaneously or sequentially. However, the latter case remains largely to be investigated. BCV may inspire this investigation, as it predicts that a higher value will be attributed to an option after presentation of lower value options. This because BCV proposes a sequential belief updating in which options considered so far contextualize the option observed now. Other predictions involve interactions regarding between- and within-choice effects. For example, consider the example above in which an agent usually evaluates equally car A (expensive and high quality) and car B (cheap and low quality). One may design an experiment where participants are first exposed to a set of cars having a fixed level of quality and varying on price. BCV predicts that this manipulation would determine a lower reward uncertainty for quality compared to price. In other words, quality would become more salient than price, predicting a preference for car A over car B. In addition, BCV predicts other forms of interactions regarding between- and within-choice effects dependent on manipulations of the reward uncertainty and the prior mean (see above), which also remain to be explored empirically. Finally, BCV may be relevant for research on the neural underpinnings of decision-making. A main aspect of this theory is the idea that incentive value corresponds to a precision-weighted reward prediction error. Interestingly, reward prediction error is reflected in activity of brain regions involved in reward processing [73]. BCV raises the possibility that a stimulus which elicits a stronger prediction error response in the brain will be attributed a higher incentive value.

There are shortcomings to BCV, though we argue that the same framework may be fruitfully used to address some of these shortcomings. A shortcoming of our current formulation assumes that model parameters are given. In reality, these parameters need to be learned in the first place. Questions about the mechanisms that might underpin learning of generative models adopted for Bayesian inference are still largely open, though substantial contributions exist, particularly in the context of structure learning [74–80]. A second shortcoming is that here we have assumed that choices occur after inference has considered all observations. An important extension of BCV is a consideration that action tendencies actually develop during evidence accumulation, and this speaks to models of choice that focus on action dynamics, sequential policy optimisation and reaction times [16, 46, 47]. Another important extension of BCV would be to generalize to domains outside incentive value computation. Context effects similar to those observed in value-based decision-making have been reported in many other conditions during perception and judgement [81–84]. Notably, multi-attribute context effects have been recently shown outside incentive value computation [85, 86], suggesting that they may derive from a general way in which the brain works [61].

### Conclusions

We offer BCV as a unifying theory of contextual effects during choice behaviour based on Bayesian normative principles. BCV predictions are in line with available empirical evidence about context sensitivity seen empirically both within and between-choice. These different effects are explained using the same simple set of principles, invoking minimal assumptions. We argue that strengths of this model are its foundation on normative principles, simplicity, the link with other influential models of brain function, and the ability to explain a wide range of empirical data. This theory may help clarify the nature of incentive value attribution and choice behaviour. This is particularly prescient when trying to understand ecological phenomena and psychopathologies characterized by dysfunctional choice, such as addiction.

## Materials and methods

Here we derive Eq 17 from the generative model shown in Fig 9B. A higher-level contextual variable (e.g., a neighbourhood containing several restaurants) is represented by a Gaussian distribution with mean *μ*_*HC*_ equal to zero and uncertainty $\sigma_{HC}^{2}$, from which a value HC is sampled. Sensory evidence about HC is provided and represented by HO which is sampled from a Gaussian distribution with mean HC and uncertainty $\sigma_{HO}^{2}$. A lower-level contextual variable (e.g., one of the restaurants) is represented by a (Gaussian) distribution with mean HC and uncertainty $\sigma_{LC}^{2}$, from which a value LC is sampled. Sensory evidence about LC is provided and represented by LO, which is sampled from a Gaussian distribution with mean LC and uncertainty $\sigma_{LO}^{2}$. A reward is obtained and sampled from a Gaussian distribution with mean LC and uncertainty $\sigma_{R}^{2}$. The posterior distribution P(LC|HO,LO,R) can be inferred sequentially in the order P(HC|HO), P(LC|HO), P(LC|HO,LO), and P(LC|HO,LO,R). The posterior mean of P(HC|HO) is:

$${\hat{\mu }}_{HC|H0}=\frac{\sigma_{HC}^{2}}{\sigma_{HC}^{2}+\sigma_{HO}^{2}}HO$$ (18)

And the posterior uncertainty:

$${\hat{\sigma }}_{HC|H0}^{2}=\sigma_{HC}^{2}-\frac{\sigma_{HC}^{2}}{\sigma_{HC}^{2}+\sigma_{HO}^{2}}\sigma_{HC}^{2}$$ (19)

The posterior mean of P(LC|HO) is equal to ${\hat{\mu }}_{HC|H0}$ (${\hat{\mu }}_{LC|H0}={\hat{\mu }}_{HC|H0}$), while the posterior uncertainty is:

$${\hat{\sigma }}_{LC|H0}^{2}={\hat{\sigma }}_{HC|H0}^{2}+\sigma_{LC}^{2}$$ (20)

The posterior mean of P(LC|HO,PO) is:

$${\hat{\mu }}_{LC|HO,LO}={\hat{\mu }}_{LC|H0}+\frac{{\hat{\sigma }}_{LC|HO}^{2}}{{\hat{\sigma }}_{LC|HO}^{2}+\sigma_{LO}^{2}}(LO-{\hat{\mu }}_{LC|H0})$$ (21)

And the posterior uncertainty:

$${\hat{\sigma }}_{LC|H0,LO}^{2}={\hat{\sigma }}_{LC|H0}^{2}-\frac{{\hat{\sigma }}_{LC|HO}^{2}}{{\hat{\sigma }}_{LC|HO}^{2}+\sigma_{LO}^{2}}{\hat{\sigma }}_{LC|H0}^{2}$$ (22)

The posterior mean of P(LC|HO,LO,R) is:

$${\hat{\mu }}_{LC|HO,LO,R}={\hat{\mu }}_{LC|HO,LO}+\frac{{\hat{\sigma }}_{LC|HO,LO}^{2}}{{\hat{\sigma }}_{LC|HO,LO}^{2}+\sigma_{R}^{2}}(R-{\hat{\mu }}_{LC|HO,LO})$$ (23)

Finally, with few rearrangements, we obtain the following incentive value for a reward offer:

$$V(R)=\frac{{\hat{\sigma }}_{LC|HO,LO}^{2}}{{\hat{\sigma }}_{LC|HO,LO}^{2}+\sigma_{R}^{2}}\left(R-\frac{{\hat{\sigma }}_{LC|HO}^{2}}{{\hat{\sigma }}_{LC|HO}^{2}+\sigma_{LO}^{2}}LO-\frac{\sigma_{LO}^{2}}{{\hat{\sigma }}_{LC|HO}^{2}+\sigma_{LO}^{2}}\frac{\sigma_{HC}^{2}}{\sigma_{HC}^{2}+\sigma_{HO}^{2}}HO\right)$$ (24)

This equation implements three normalization factors: (i) a subtractive normalization factor $\left(\frac{{\hat{\sigma }}_{LC|HO}^{2}}{{\hat{\sigma }}_{LC|HO}^{2}+\sigma_{LO}^{2}}LO\right)$ proportional to the value LO observed at the low contextual level, (ii) a subtractive normalization factor $\left(\frac{\sigma_{LO}^{2}}{{\hat{\sigma }}_{LC|HO}^{2}+\sigma_{LO}^{2}}\frac{\sigma_{HC}^{2}}{\sigma_{HC}^{2}+\sigma_{HO}^{2}}HO\right)$ proportional to the value HO observed at the high contextual level, (iii) a divisive normalization factor $\left(\frac{{\hat{\sigma }}_{LC|HO,LO}^{2}}{{\hat{\sigma }}_{LC|HO,LO}^{2}+\sigma_{R}^{2}}\right)$ that captures the weighting dependent on the (relative) reward uncertainty. If we define the three factors as *τ*_*LO*_ and *τ*_*HO*_ and K respectively, we obtain Eq 17.
