# Correction: A unifying Bayesian account of contextual effects in value-based choice

**DOI:** 10.1371/journal.pcbi.1007366

**Published:** 2019-10-02

**Authors:** Francesco Rigoli, Christoph Mathys, Karl J. Friston, Raymond J. Dolan

Fig 5 and Fig 6 are incorrect. The authors have provided a corrected version here.

**Fig 5:**
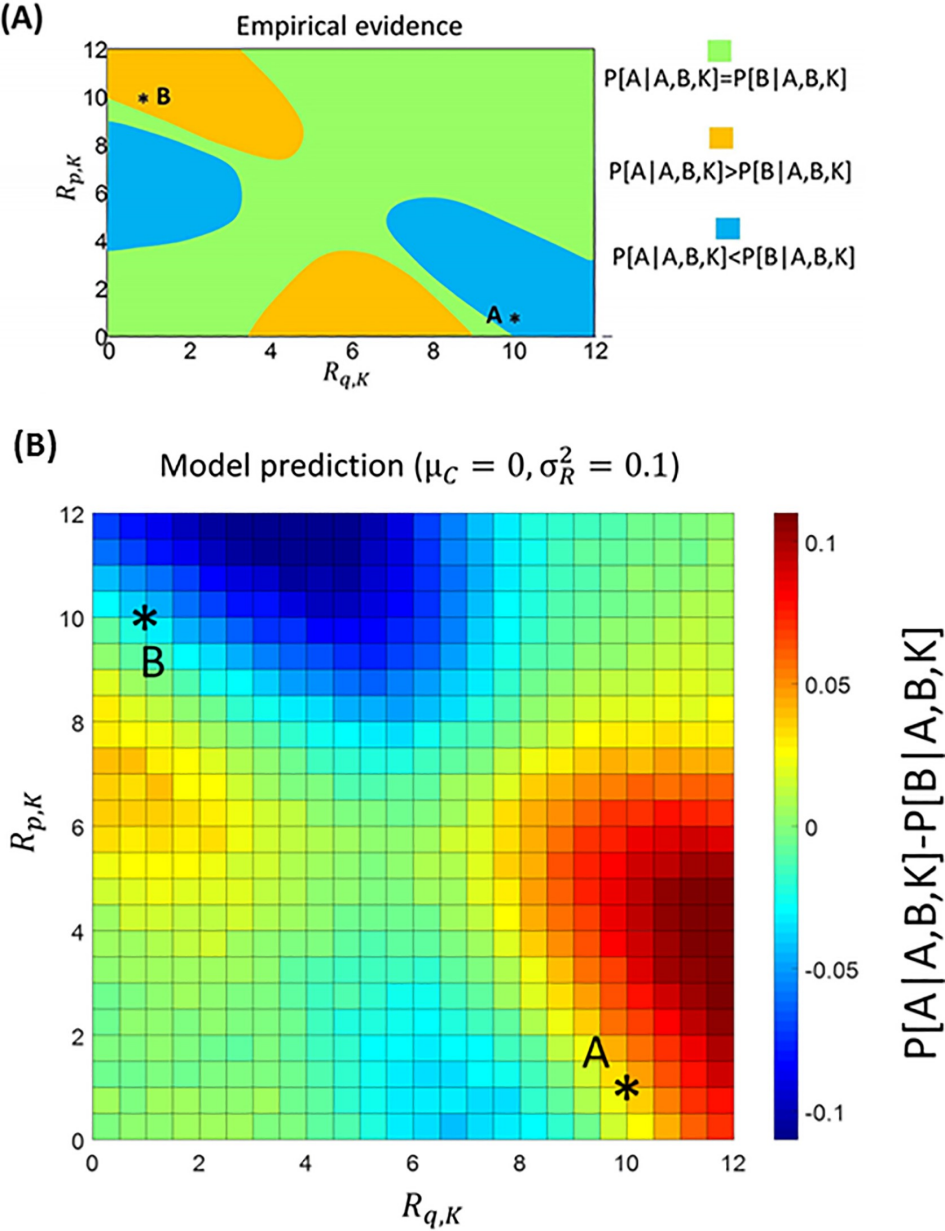
**A** Empirical evidence (derived from integrating data from available studies as in [19]) concerning the difference in probability between choosing option A and option B when a third option K is available (*P*[*A*|*A*,*B*,*K*] − *P*[*B*|*A*,*B*,*K*]). Here options are characterized by two attributes (price *p* and quality *q*). For car A, we assign *R*_*p*,*A*_ = 1 to price (low scores indicate high price) and *R*_*q*,*A*_ = 10 to quality. For car B, we assign *R*_*p*,*B*_ = 10 to price and *R*_*q*,*B*_ = 1 to quality. The graph considers the choice probability difference between option A and option B as a function of the reward amounts *R*_*q*,*K*_ (for quality; x axis) and *R*_*p*,*K*_ (for price; y axis) of a third option K. Green areas indicate values for which no difference is expected based on empirical evidence; orange and blue areas indicates values for which a positive and negative difference is expected, respectively. **B:** The same analysis is performed with data simulated using BCV (100000 trials are simulated for each condition; μ_*C*_ = 0; $\sigma_{R}^{2}=0.1$; $\sigma_{C}^{2}$ = 1 for simulations).

**Fig 6:**
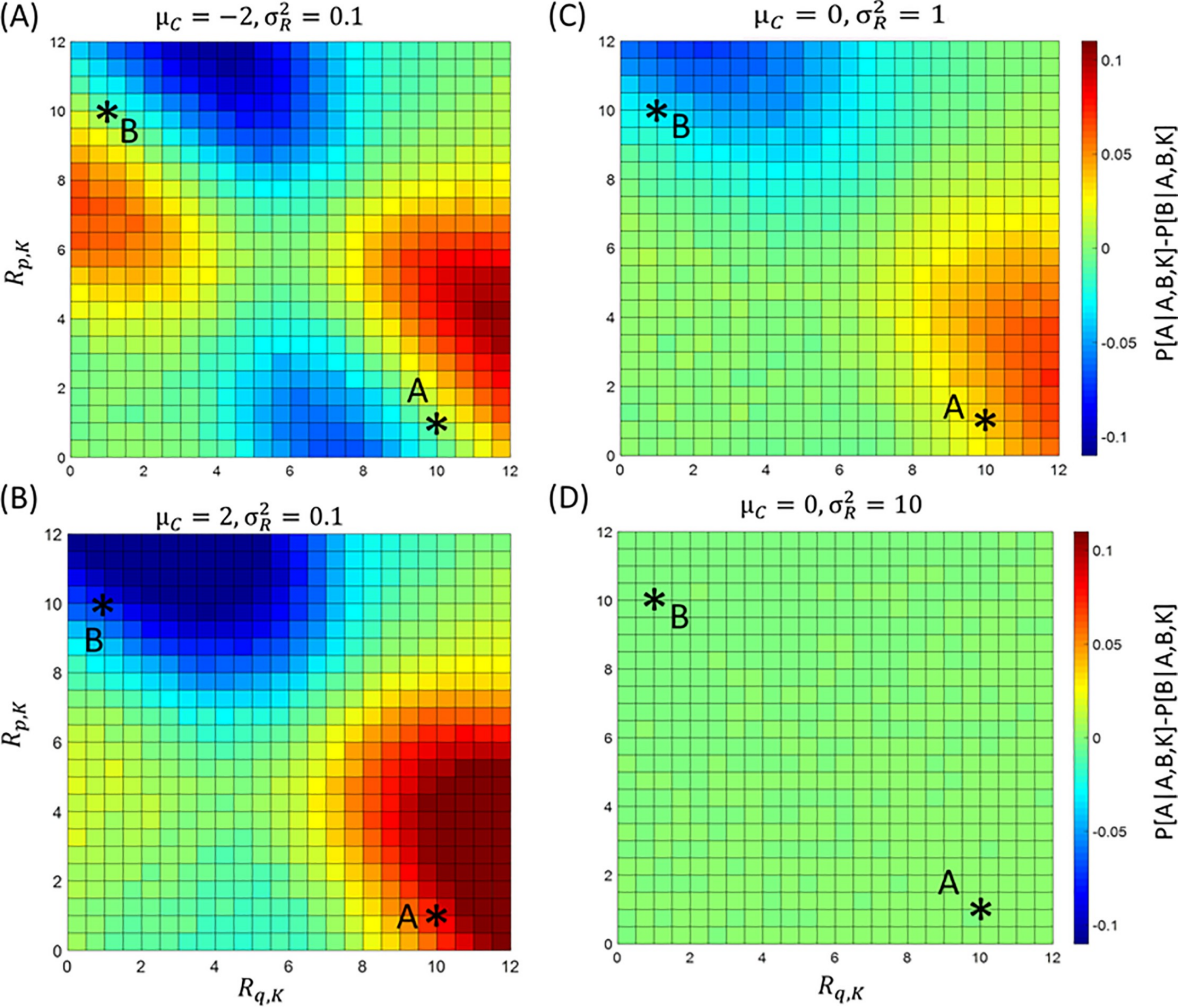
**Predictions of BCV about the difference in probability between choosing option A and option B when a third option K is available (*P*[*A*|*A*,*B*,*K*] − *P*[*B*|*A*,*B*,*K*]).** Here options are characterized by two attributes (price *p* and quality *q*). For car A, we assign *R*_*p*,*A*_ = 1 to price (low scores indicate high price) and *R*_*q*,*A*_ = 10 to quality. For car B, we assign *R*_*p*,*B*_ = 10 to price and *R*_*q*,*B*_ = 1 to quality. The graph considers the choice probability difference between option A and option B as a function of the reward amounts *R*_*q*,*K*_ (for quality; x axis) and *R*_*p*,*K*_ (for price; y axis) of a third option K (100000 trials are simulated for each condition; $\sigma_{C}^{2}$ = 1 for simulations). Different parameter sets are swn. **A:** Simulation using μ_*C*_ = −2 and $\sigma_{R}^{2}=0.1$. **B:** Simulation using μ_*C*_ = 2 and $\sigma_{R}^{2}=0.1$. **C:** Simulation using μ_*C*_ = 0 and $\sigma_{R}^{2}=1$. **D:** Simulation using μ_*C*_ = 0 and $\sigma_{R}^{2}=10$.
